# Ultra-Wideband Impulse Radar Through-Wall Detection of Vital Signs

**DOI:** 10.1038/s41598-018-31669-y

**Published:** 2018-09-06

**Authors:** Xiaolin Liang, Jianqin Deng, Hao Zhang, Thomas Aaron Gulliver

**Affiliations:** 1 Science and Technology on Electronic Test & Measurement Laboratory, The 41st Research Institute of CETC, Xiang Jiang Road 98th, Qingdao, People’s Republic of China; 2 Department of Electronic Engineering, Ocean University of China, Song Ling Road 238th, Qing Dao, People’s Republic of China; 3 Department of Electrical Computer Engineering, University of Victoria, PO Box 1700, STN CSC, Victoria, BC V8W 2Y2 Canada

## Abstract

This paper presents a new system for the detection of human respiration behind obstacles using impulse ultra-wideband (UWB) radar. In complex environments, low signal-to-noise ratios (SNRs) as they can result in significant errors in the respiration, heartbeat frequency, and range estimates. To improve the performance, the complex signal demodulation (CSD) technique is extended by employing the signal logarithm and derivative. A frequency accumulation (FA) method is proposed to suppress mixed products of the heartbeat and respiration signals and spurious respiration signal harmonics. The respiration frequency is estimated using the phase variations in the received signal, and a discrete short-time Fourier transform (DSFT) is used to estimate the range. The performance of the proposed system is evaluated along with that of several well-known techniques in the literature.

## Introduction

In recent years, the detection of living persons behind obstacles impulse ultra-wideband (UWB) radar has been investigated^1–5^. UWB radar has been used for the detection of humans^6,7^, moving subjects^8,9^, imaging in through-wall conditions^10,11^, search and rescue^12,13^, positioning indoors^14,15^, and public order and security^16^ because of its high range resolution and penetrability^17–20^. It can be used to detect human vital sign signals such as respiration and heart rates, but this can be difficult as thorax movement is usually only several millimeters, and the signal attenuation can be severe.

Previous researches on human vital sign detection have focused on suppressing stationary and/or nonstationary clutters, estimating the respiration frequency and heart rate, the analysis of signal characteristics, and other related problems^21–42^. The characteristics of human respiration signals were analyzed in both time and frequency domain^24,25^ using the Hilbert-Huang transform (HHT) and fast Fourier transform (FFT). In^27^, clutter similar to respiration signals was suppressed using adaptive clutter cancellation. In^28^, the arctangent demodulation (AD) technique was employed in a UWB pulse radar system to accurately extract vital sign signals over long distances and in and through-wall conditions. However, this technique is complex and decreases the computational efficiency. A maximum likelihood estimator was considered in^29^ to determine the period of vital sign signals under considering the additive-white-Gaussian-noise (AWGN). A post-processing algorithm was developed for respiration motion detection. The stationary and nonstationary clutters were removed by employing the singular-value-decomposition (SVD) algorithm when the signal-to-noise ratio (SNR) is low^30^. A tracing technique was considered to extract the respiration impulse response, but this approach is effective only when the SNR is high and over small distances^35^. LTS i.e. linear trend subtraction method was employed in^30^ to reduce the linear trend. In^37^, a higher order cumulants (HOC) technique was applied to extract vital sign signals considering that the fourth order cumulants of AWGN is zero. The classic EEMD i.e. ensemble empirical mode decomposition technique was used in^38^ to estimate the heart rate of a living person by improving the SNR and removing clutter. An improved arctangent demodulation (AD) algorithm was proposed to increase the accuracies of human heart rate estimate^40^. In^42^, a state-space method (SSM) was employed to extract vital sign signals including human respiration and heartbeat signals.

Most detection techniques are not effective over long distances and in through-wall conditions. To solve these problems, an algorithm based on UWB radar is developed here to calculate accurately vital sign signals in challenging environments. This is achieved by suppressing stationary, non-stationary and other clutter as well as the linear trend. The products of the vital sign signals and their harmonics are removed by employing a CSD-based frequency accumulation (FA) method. This is shown to perform better than several well-known techniques. The range information between human subject and radar is estimated considering the characteristics of vital sign signals and a discrete short-time Fourier transform (DSFT). Experimental results obtained using a UWB radar system at the Key Laboratory of Electromagnetic Radiation and Sensing Technology, Institute of Electronics, Chinese Academy of Sciences (IECAS), are used to evaluate the performance of the proposed technique.

The following parts of the paper are arranged as follows. The model of signal detection is given in Section 2, and the proposed method for vital sign detection is presented in Section 3. Section 4 provides performance results to evaluate the detection method, and the conclusions are presented in Section 5.

## Signal Detection

### Vital Sign Signal Model

Vital sign signals may be obtained due to the time delay changes of a transmitted UWB pulse. The distance between human chest and radar is^32^

1 $$d(t)={d}_{0}+r(t)={d}_{0}+{A}_{r}\,\sin (2\pi {f}_{r}t)+{A}_{h}\,\sin (2\pi {f}_{h}t),$$

where the range between the radar and the center of human chest is given by *d*_0_^30^, *f*_*r*_ and *A*_*r*_ are the frequency and amplitude of human respiratory movement, respectively. ${f}_{h}$ and ${A}_{h}$ are the heart rate and heartbeat amplitude, respectively^32^.

With only one human subject present in the detection environment and all other objects are considered as static, the impulse responses are

2 $$h(\tau ,\,t)={a}_{v}\delta (\tau -{\tau }_{v}(t))+\sum _{i}{a}_{i}\delta (\tau -{\tau }_{i}),$$

where $\sum _{i}{a}_{i}\delta (\tau -{\tau }_{i})$ represent the responses from all these static objects with time delay *τ*_*i*_ and vibration amplitude *a*_*i*_^30^, and ${a}_{v}\delta (\tau -{\tau }_{v}(t))$ represents the impulse response of the vital signs with amplitude *a*_*v*_ and time delay ${\tau }_{v}(t)$^32^. This delay is then

3 $${\tau }_{v}(t)=\frac{2d(t)}{v}={\tau }_{0}+{\tau }_{r}\,\sin (2\pi {f}_{r}t)+{\tau }_{h}\,\sin (2\pi {f}_{h}t),$$

where $$v=3\times {10}^{8}$$ m/s, $${\tau }_{0}=2{d}_{0}/v$$,$${\tau }_{r}=2{A}_{r}/v$$, and $${\tau }_{h}=2{A}_{h}/v$$.

**Figure 1:**
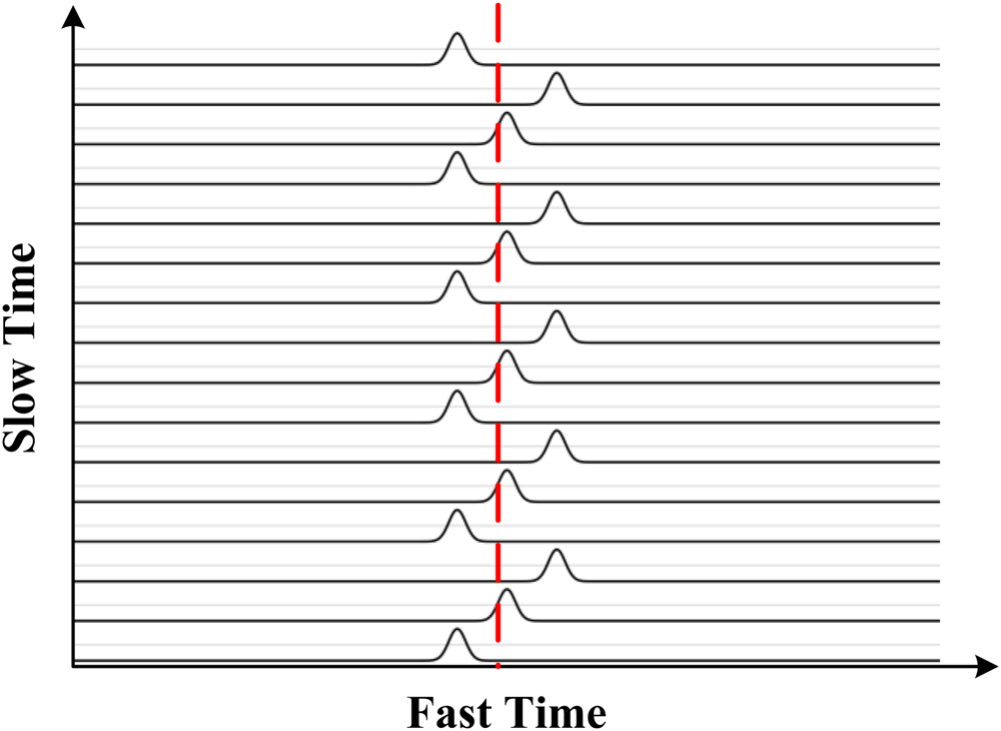
An illustration of the received pulses due to human respiration only.

The signal received at the radar is then

4 $$R(\tau ,\,t)=s(\tau )\ast h(t,\,\tau )={a}_{v}s(\tau -{\tau }_{v}(t))+\sum _{i}{a}_{i}s(\tau -{\tau }_{i}),$$

where $s(\tau )$ represents the transmitted pulse. $R(\tau ,t)$ for a respiration signal is shown in Fig. 1. Slow-time represents the received pulse number and fast-time represents the range. In the signal model, *t* is slow-time with frequency components denoted by *f*, and *τ* is fast-time with frequency components denoted by *υ*. The average human chest location is shown as a dashed line.

The discrete signal corresponding to (4) is

5 $$\begin{array}{rcl}R[m,n] & = & {a}_{v}s(m{\delta }_{T}-{\tau }_{v}(n{T}_{s}))+\sum _{i}{a}_{i}s(m{\delta }_{T}-{\tau }_{i})\\  & = & {a}_{v}s(m{\delta }_{R}-v{\tau }_{v}(n{T}_{s}))+\sum _{i}{a}_{i}s(m{\delta }_{R}-v{\tau }_{i}/2)\\  & = & h[m,\,n]+c[m],\end{array}$$

where $1/{T}_{s}$ represents the pulse repetition frequency (PRF), $t=n{T}_{s}$, $n=0,\ldots ,\,N-1$ denote *t* samples, *δ*_*T*_ represents the sampling time in *τ*, ${\delta }_{R}=v{\delta }_{T}/2$ $m=0,\ldots ,\,M-1$ denote *τ* samples, $h[m,n]$ represent vital signs, and $c[m]$ represent static clutters. To avoid ambiguities in range and frequency aliasing, *T*_*s*_ should satisfy

6 $$1/{T}_{s}\ge 2(\max ({f}_{r},\,{f}_{h})).$$

In a real environment, (5) becomes

7 $${R}_{M\times N}={h}_{M\times N}+{c}_{M\times N}+{a}_{M\times N}+{w}_{M\times N}+{q}_{M\times N}+{g}_{M\times N}+{z}_{M\times N},$$

where ${a}_{M\times N}$ represents linear trend term, ${w}_{M\times N}$ represents AWGN, ${q}_{M\times N}$ represents non-stationary clutters, ${g}_{M\times N}$ represents unknown clutters, and ${z}_{M\times N}$ represents the clutters introduced by moving objects. A reflected pulse must be acquired within one pulse duration so that

8 $${T}_{w}+\,\max \,\{{\tau }_{v}(t)\}-\,\min \,\{{\tau }_{v}(t)\} < {T}_{s},$$

where *T*_w_ is the −6 dB pulse width.

### Vital Sign Signal Detection

In static environments, the received pulses are shown in (4). Usually, the pulses without $c[m]$ are

9 $$\tilde{R}(\tau ,\,t)={a}_{v}s(\tau -{\tau }_{v}(t)).$$

The frequencies of the vital sign signals are obtained by performing FT on $\tilde{R}({\rm{m}}{\delta }_{T},t)$ in *t* given by

10 $$Y(m{\delta }_{T},\,f)={\int }_{-\infty }^{+\infty }\tilde{R}(m{\delta }_{T},\,t){e}^{-j2\pi ft}dt.$$

This can be acquired using the two-dimension FT of $Y(\upsilon ,f)$ given by

11 $$Y(m{\delta }_{T},\,f)={\int }_{-\infty }^{+\infty }Y(\upsilon ,\,f){e}^{j2\pi \upsilon \tau }d\upsilon ,$$

where

12 $$Y(\upsilon ,\,f)={\int }_{-\infty }^{+\infty }{\int }_{-\infty }^{+\infty }\tilde{R}(m{\delta }_{T},\,t){e}^{-j2\pi ft}{e}^{-j2\pi \upsilon \tau }dtd\tau ,$$

13 $$\begin{array}{rcl}Y(\upsilon ,f) & = & {\int }_{-\infty }^{+\infty }{a}_{v}S(\upsilon ){e}^{-j2\pi ft}{e}^{-j2\pi \upsilon {\tau }_{v}(t)}dt,\\  & = & {a}_{v}S(\upsilon ){e}^{-j2\pi \upsilon {\tau }_{0}}{\int }_{-\infty }^{+\infty }{e}^{-j2\pi \upsilon {m}_{b}\sin (2\pi {f}_{r}t)}{e}^{-j2\pi \upsilon {m}_{h}\sin (2\pi {f}_{h}t)}{e}^{-j2\pi ft}dt,\end{array}$$

and $S(\upsilon )$ is the FT of the transmitted UWB pulse. This can be represented using Bessel functions as

14 $$\begin{array}{rcl}Y(\upsilon ,\,f) & = & {a}_{v}S(\upsilon ){e}^{-j2\pi \upsilon {\tau }_{0}}\\  &  & \times \,{\int }_{-\infty }^{+\infty }(\sum _{k=-\infty }^{+\infty }{J}_{k}({\beta }_{r}\upsilon ){e}^{-j2\pi k{f}_{r}t})(\sum _{l=-\infty }^{+\infty }{J}_{l}({\beta }_{h}\upsilon ){e}^{-j2\pi l{f}_{b}t}){e}^{-j2\pi ft}dt.\end{array}$$

15 $${e}^{-jz\sin (2\pi {f}_{0}t)}=\sum _{k=-\infty }^{+\infty }{J}_{k}(z){e}^{-j2\pi k{f}_{0}t}.$$

where $${\beta }_{r}=2\pi {A}_{r}$$ and $${\beta }_{h}=2\pi {A}_{h}$$.

Then, (10) is rewritten as

16 $$Y(m{\delta }_{T},\,f)={a}_{v}\sum _{k=-\infty }^{+\infty }\sum _{l=-\infty }^{+\infty }{G}_{kl}(\tau )\delta (f-k{f}_{r}-l{f}_{h}).$$

where

17 $${G}_{kl}(\tau )={\int }_{-\infty }^{+\infty }S(\upsilon ){J}_{k}({\beta }_{r}\upsilon ){J}_{l}({\beta }_{h}\upsilon ){e}^{j2\pi \upsilon (\tau -{\tau }_{0})}d\upsilon .$$

The maximum value of (17) can be obtained as

18 $${C}_{kl}={G}_{kl}({\tau }_{0})={\int }_{-\infty }^{+\infty }S(\upsilon ){J}_{k}({\beta }_{r}\upsilon ){J}_{l}({\beta }_{h}\upsilon )d\upsilon $$

and setting $l=0$ in

19 $$Y({\tau }_{0},\,f)={a}_{v}\sum _{k=-\infty }^{+\infty }\sum _{l=-\infty }^{+\infty }{C}_{kl}\delta (f-k{f}_{r}-l{f}_{h}),$$

gives the respiration signal

20 $${C}_{k{\rm{0}}}={\int }_{-\infty }^{+\infty }S(\upsilon ){J}_{k}({\beta }_{r}\upsilon ){J}_{{\rm{0}}}({\beta }_{h}\upsilon )d\upsilon .$$

setting *k* = 0 in (19), we can acquire the heartbeat signals as

21 $${C}_{0l}={\int }_{-\infty }^{+\infty }S(\upsilon ){J}_{0}({\beta }_{b}\upsilon ){J}_{l}({\beta }_{h}\upsilon )d\upsilon \approx {\int }_{-\infty }^{+\infty }S(\upsilon ){J}_{l}({\beta }_{h}\upsilon )d\upsilon .$$

### UWB Radar

The experimental data was obtained using a UWB radar with parameters given in^32^. The radar center frequency is 400 MHz and the PRF is 600 kHz. The UWB pulses were acquired form six segments simultaneously with a time window of 124 ns, *M*_*s*_ = 682 samples per segment, and *M* = 4092 fast-time samples. *N*_*A*_ samples are averaged to improve the SNR. Thus, a set of data is obtained every *M*_*s*_*N*_*A*_/PRF = 34.1 ms, and it takes 17.6 s to receive *N* = 512 pulses in slow-time. A combination of equivalent-time and real-time sampling^34^ is employed as it provides better performance than equivalent-time sampling only^35^.

**Figure 2:**
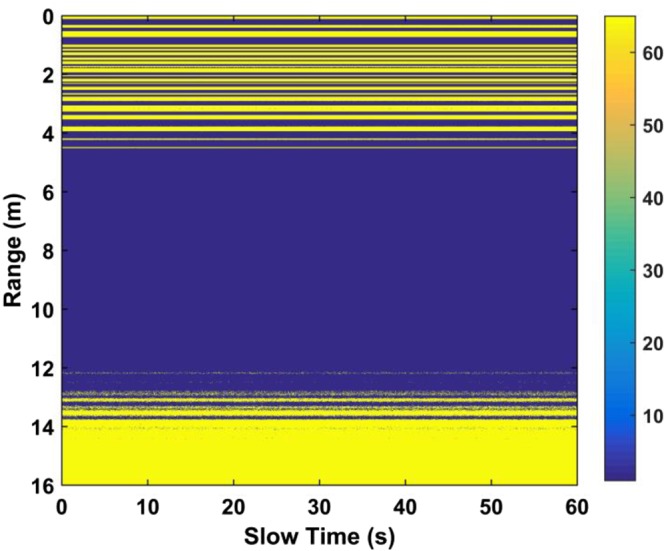
The received UWB radar signal.

Figure 2 shows the matrix *R* obtained using the UWB radar with a human subject at a distance of 9 m from the receiver in an outdoor environment which is described in Section IV. The vital sign signals are not evident due to the significant signal attenuation caused by the through-wall and long distance conditions. Thus, obtaining these signals in real environments is challenging, which motivates the development of a new method for vital sign detection.

## Detection Algorithm

**Figure 3:**
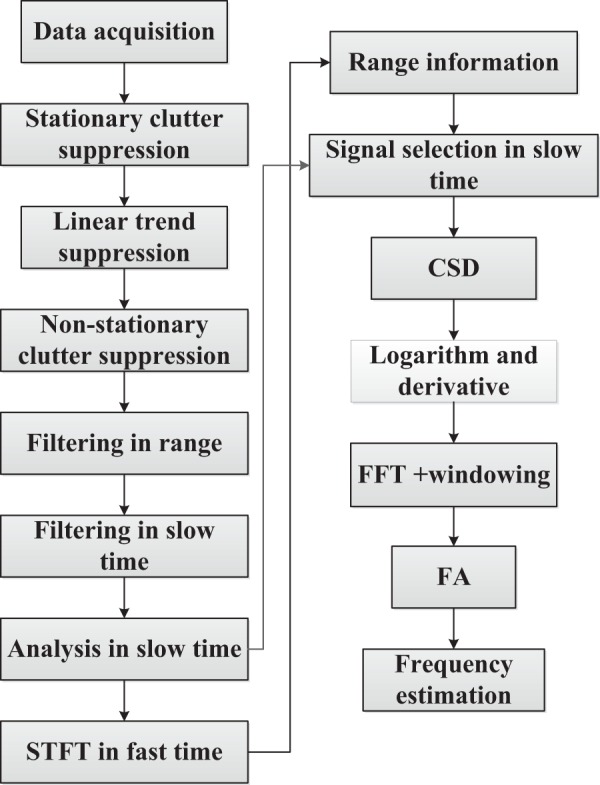
Flowchart of the proposed detection algorithm.

The new method for detection of the vital sign is designed in this section. A flowchart of this algorithm is given in Fig. 3. Two volunteers participated in this research. They were informed of the risks associated with participating in the experiments. This research was approved by both Ocean University of China and the Chinese Academy of Sciences. All experiments were performed in accordance with the relevant international guidelines and regulations.

### Clutter Suppression

Respiration signals are typically corrupted by strong static clutters which are approximately slow-time invariant^30^. The DC component can be estimated as

22 $$\Im =\frac{1}{M\times N}\sum _{m=1}^{M}\sum _{n=1}^{N}R[m,\,n]$$

resulting in the matrix^31^

23 $${{\rm{\Omega }}}_{M\times N}={{\rm{\Psi }}}_{M\times N}-\Im $$

To further remove static clutter, range profile subtraction is employed which is^32^

24 $$\widehat{{\rm{\Omega }}}[m,n]={\rm{\Omega }}[m,n]-{\rm{\Omega }}[m,\,n-1]$$

and then adaptive background subtraction is applied using a weighting coefficient $\lambda$ to obtain

25 $$p[m,\,n]=\lambda p[m,\,n-1]+(1-\lambda ){\rm{\Omega }}[m,\,n]$$

which gives

26 $$T[m,\,n]={\rm{\Omega }}[m,\,n]-p[m,\,n]$$

where $$\lambda =0.9$$ and $$p[m,1]={\rm{\Omega }}[m,1]$$^43^.

The LTS method estimates the linear trend ${a}_{M\times N}$ and removes it from the received pulses. A least-squares fit is used to eliminate ${a}_{M\times N}$ from the received pulses in slow-time by assuming that the starting point is a linear model for the received pulses. This can be expressed in matrix form as

27 $$T=X\beta $$

where $X=[\frac{k}{N}\,{1}_{N}]$, $k=0,1,\ldots ,N-1,$ and $\beta$ denotes the unknown coefficient matrix which can be obtained by multiplying (27) by ${X}^{{\rm{T}}}$

28 $${X}^{{\rm{T}}}T=({X}^{{\rm{T}}}X)\beta $$

Rearranging gives

29 $${({X}^{{\rm{T}}}X)}^{-1}{X}^{{\rm{T}}}T=\beta $$

Using (28) and (29), the linear trend can be calculated as

30 $$a=X\beta =X{({X}^{{\rm{T}}}X)}^{-1}{X}^{{\rm{T}}}T$$

As a result, we can acquire the results given by

31 $$W=T-X{({X}^{{\rm{T}}}X)}^{-1}{X}^{{\rm{T}}}T$$

In high SNR conditions, vital signs can easily be obtained even under ${q}_{M\times N}$ conditions. However, this clutter can significantly degrade signal estimation. SVD divides $W$ into complementary subspaces which represent independent features of the received signal. This can be used to reduce the static/non-static clutter and increase the SNR. This decomposition is given by

32 $$W=US{V}^{{\rm{T}}}$$

where $S$ is a diagonal matrix, $U$ is a $M\times M$ unitary matrix and $V$ is a $N\times N$ unitary matrix. The elements of $S$ are the singular values ${\sigma }_{i}$ which are ranked in descending order, i.e. $\sigma 1\ge \sigma 2\ge \sigma 3\ge \cdot \cdot \cdot \ge {\sigma }_{r}\ge 0$. The columns of $U$ are the left singular vectors and the columns of $V$ are the right singular vectors. The eigenvectors of $W{W}^{{\rm{T}}}$ are *U* and the eigenvectors of ${W}^{{\rm{T}}}W$ are *V*.

The SVD in (32) can be expressed as

33 $$\begin{array}{rcl}W & = & {\sigma }_{1}(\begin{array}{c}\cdot \\ \cdot \\ {\mu }_{1}\\ \cdot \\ \cdot \end{array})({\upsilon }_{1}\ldots )+{\sigma }_{k}(\begin{array}{c}\cdot \\ \cdot \\ {\mu }_{k}\\ \cdot \\ \cdot \end{array})(\ldots {\upsilon }_{k}\ldots )+\cdot \cdot \cdot +{\sigma }_{N}(\begin{array}{c}\cdot \\ \cdot \\ {\mu }_{N}\\ \cdot \\ \cdot \end{array})(\ldots {\upsilon }_{N}\ldots )\\  & = & {M}_{1}+{M}_{2}+\cdot \cdot \cdot +{M}_{k}+\cdot \cdot \cdot +{M}_{G}\end{array}$$

where ${M}_{k}$ is the *k*th intrinsic image with the same dimensions as $W$. The intrinsic images are arranged in descending order. *M*_1_ is the first intrinsic image and is considered as ${q}_{M\times N}$. The images *M*_2_, *M*_3_, …, *M*_*k*_ typically contain the majority of the vital sign signals, while *M*_*k*+1_,…, *M*_*G*_ mainly contain noise. Therefore, (33) can be rewritten as

34 $$W={M}_{C}+{M}_{T}+{M}_{N}$$

where ${M}_{C}$ denotes ${q}_{M\times N}$, i.e. the first intrinsic image, ${M}_{T}$ is the images containing the vital sign signals and ${M}_{N}$ is the remaining images. SVD can be used to remove ${q}_{M\times N}$ which gives

35 $${\rm{\Phi }}=US{V}^{{\rm{T}}}=\sum _{i=2}^{G}{u}_{i}{\sigma }_{ii}{v}_{i}^{{\rm{T}}}$$

Note that it is difficult to estimate *k* so it is not possible to suppress the noise using this technique.

### SNR Improvement

The detection environment including the signal frequency can have a significant effect on the received signals^37^. As a result, the traditional matched filter maybe invalid for vital signs detection. Instead, a Butterworth bandpass filter (BPF) is employed which has transfer function^44^

36 $${|H(\omega )|}^{2}=\frac{1}{1+{(\omega /{\omega }_{c})}^{2{N}_{f}}}$$

where ${N}_{f}=5$ is the filter order and ${\omega }_{c}$ is the cutoff frequency. This filter is composed of a Butterworth low pass filter (LPF) and a Butterworth high pass filter (HPF). This filter is used on (35) in the fast-time which gives

37 $$\begin{array}{rcl}{\rm{\Lambda }}[m,\,n] & = & {\chi }_{1}{\rm{\Phi }}[m,\,n]+{\chi }_{2}{\rm{\Phi }}[m-1,\,n]+\mathrm{...}+{\chi }_{{N}_{b}+1}{\rm{\Phi }}[m-{N}_{b},\,n]\\  &  & -\,{\kappa }_{2}{\rm{\Phi }}[m-1,\,n]-\cdots -{\kappa }_{{N}_{a}+1}{\rm{\Phi }}[m-{N}_{a},\,n]\end{array}$$

where ${N}_{b}={N}_{a}=5$, ${\kappa }_{i}$ and ${\chi }_{i}$ are the filter coefficients.

An average extraction filter is now used to eliminate low- and high-frequency clutter. The output is

38 $${\rm{\Psi }}[k,\,n]=\frac{1}{\beta }\sum _{m=\beta k}^{\beta (k+1)-1}{\rm{\Lambda }}[m,\,n],$$

where $k=1,\,\ldots ,\,\lfloor M/\beta \rfloor$, $\beta =7$ is the number of filter coefficients, $\lfloor M/\beta \rfloor$ is the maximum integer less than $M/\beta$, and ${{\boldsymbol{\Lambda }}}_{M\times N}=0$ when $M > \lfloor M/\beta \rfloor$. Equations (22–38) are used to suppress the components $c[m]$, ${a}_{M\times N}$, ${q}_{M\times N}$, and ${g}_{M\times N}$. ${z}_{M\times N}$ can be removed when there are no motions in the detection area. The resulting ideal signal is then

39 $$\begin{array}{rcl}{\rm{\Psi }}[m,\,n] & = & {a}_{v}s(m{\delta }_{T}-{\tau }_{v}(n{T}_{s}))\\  & = & {a}_{v}s(m{\delta }_{R}-v{\tau }_{v}(n{T}_{s}))=h[m,\,n]\end{array}$$

### Range Determination

**Figure 4:**
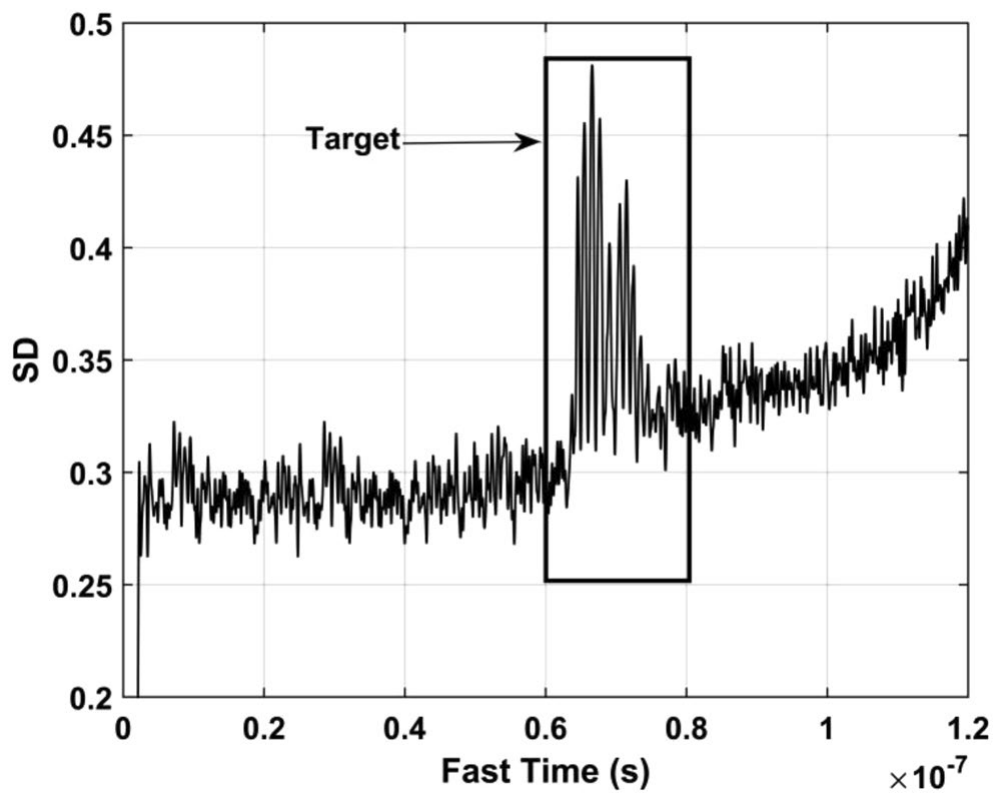
The SD obtained for a human subject outdoors located 9 m from the radar.

**Figure 5:**
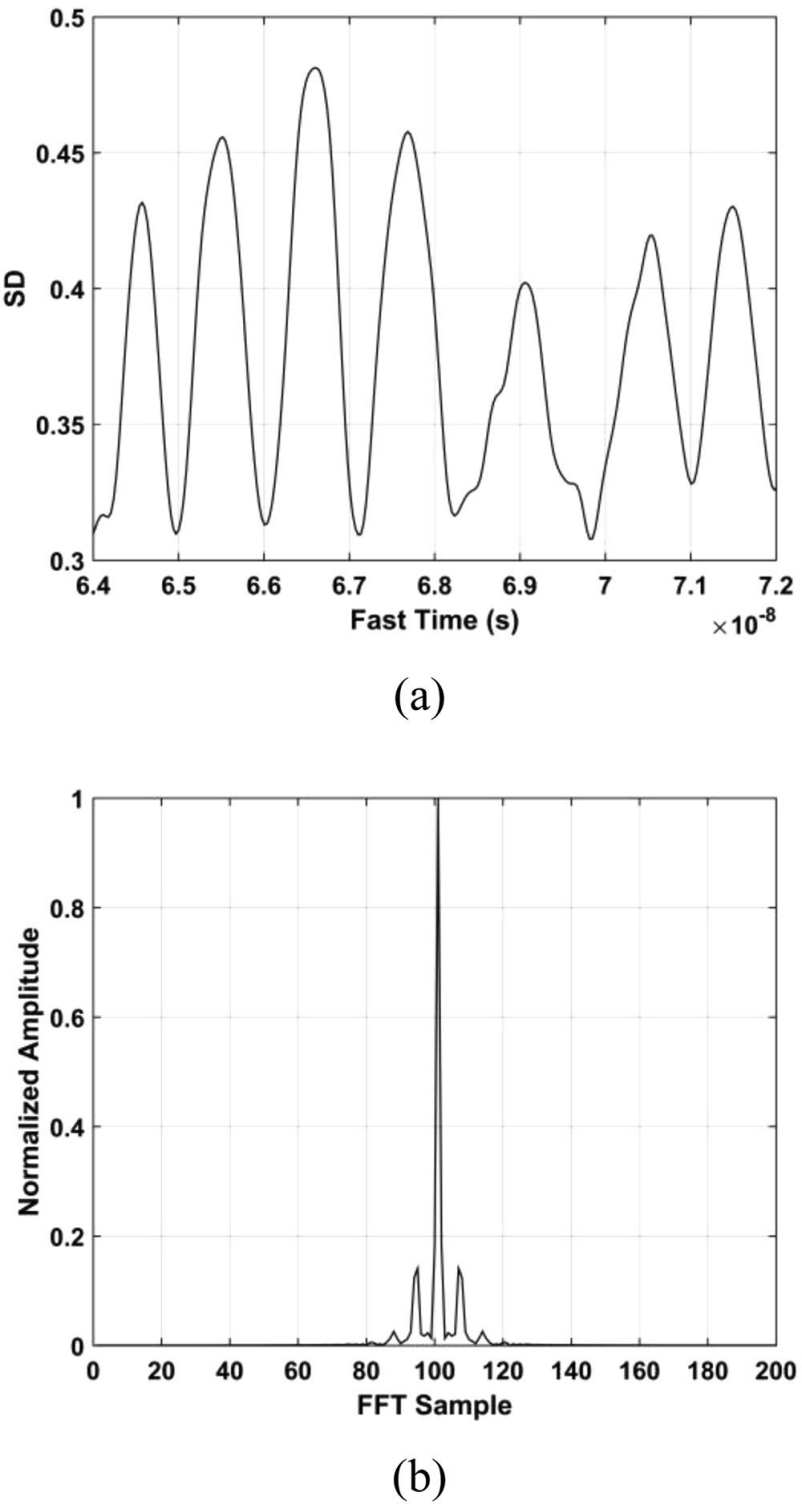
The SD in the target area in (**a**) the time-domain, and (**b**) the corresponding FFT.

The standard deviation (SD) of the received pulses is used for range estimation of human subject. The SD for fast-time index *m* is^45^

40 $${\rm{\Gamma }}[m]=\sqrt{\frac{\sum _{n=1}^{N}{({\rm{\Psi }}[m,n]-{\mu }_{m})}^{2}}{N-1}}$$

where ${\mu }_{m}$ is the mean. The data from a female volunteer at 9 m far from the radar were used for SD calculation in through-wall conditions, and Fig. 4 gives the results. This figure shows that the SD is large when a subject is present, which indicates that it can be used for the detection of vital sign signals. Figure 5 shows the corresponding spectrums obtained from the SD values in the target area using an FT, which indicates the SD is periodic, and thus can be used for range estimation.

**Figure 6:**
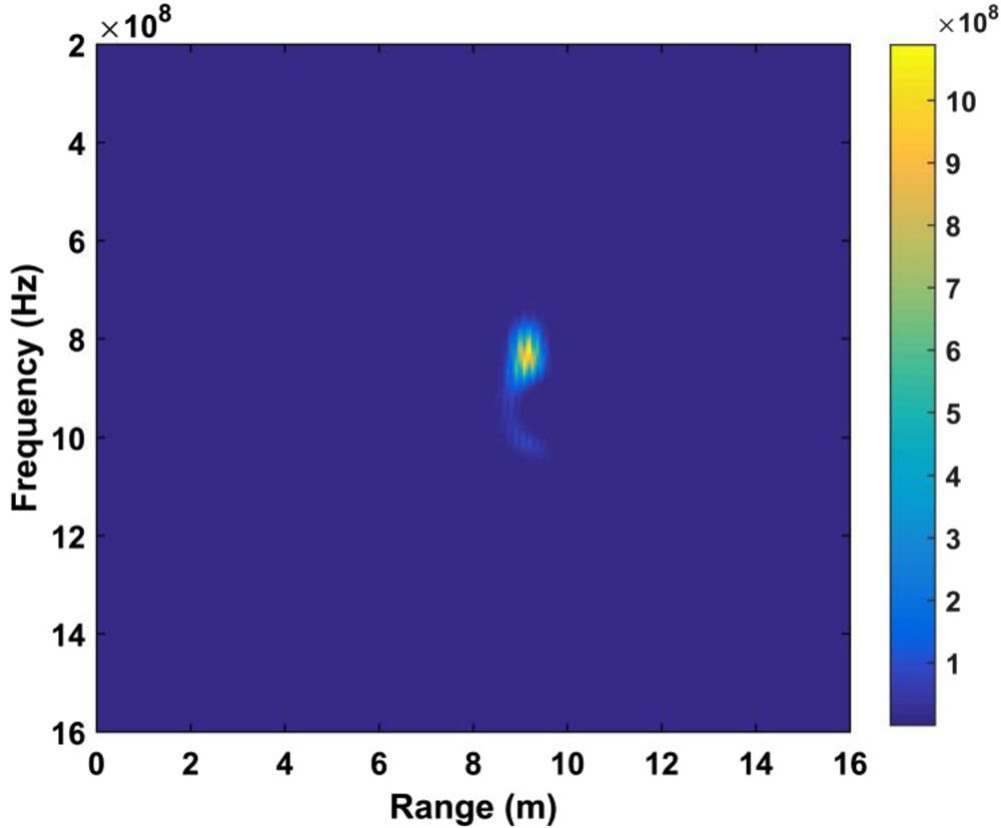
The STFT for a human subject outdoors located 9 m from the radar.

**Figure 7:**
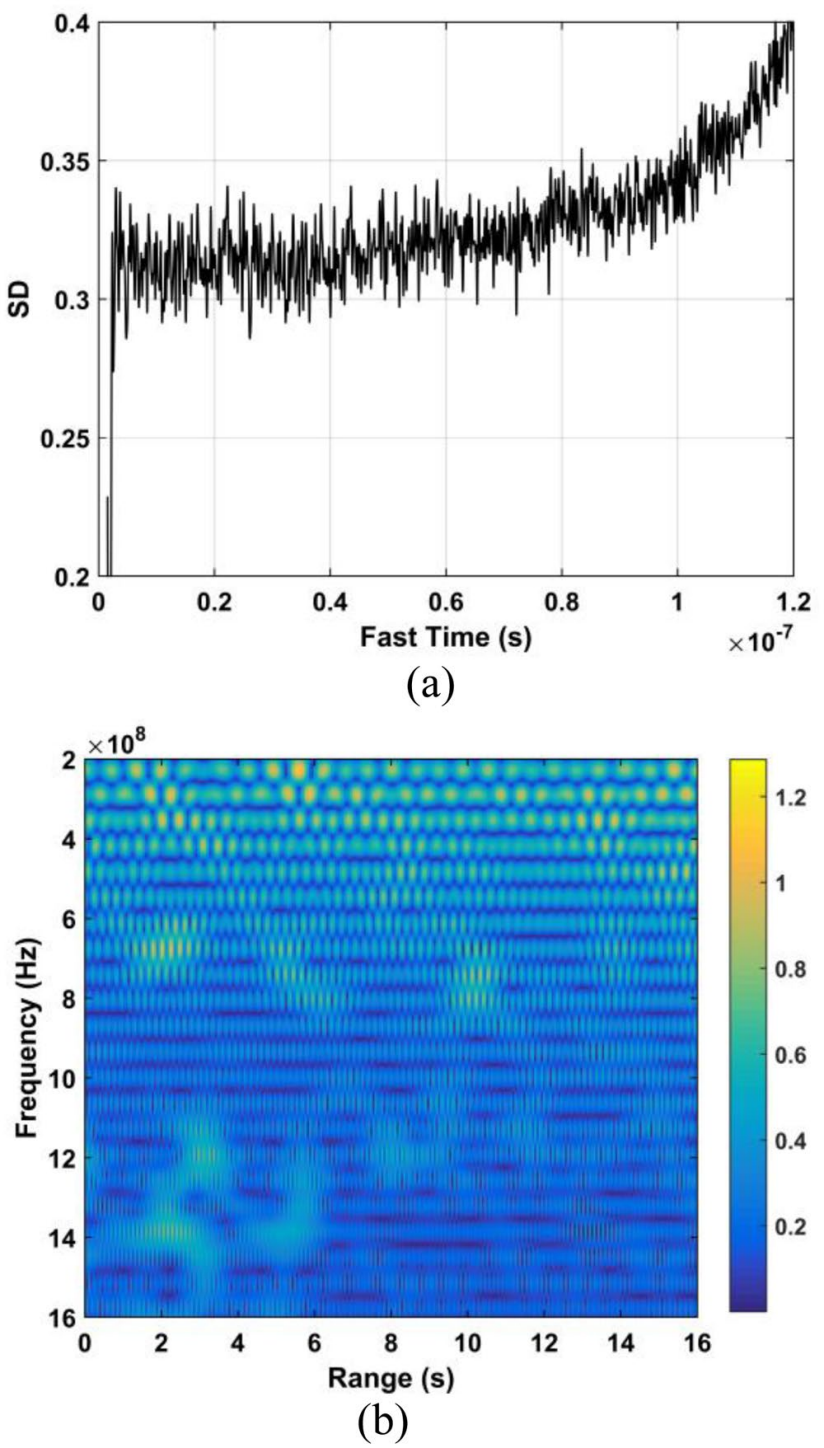
Results without a human subject (**a**) the SD, and (**b**) the corresponding STFT.

The DSFT of (40) is used for analyzing signal characteristics in time-frequency domain^46,47^. Windowing is also employed, which gives

41 $$K[o,\,p]=\sum _{m=1}^{M}{\rm{\Gamma }}[m]{\rm{\Xi }}[o-m]{e}^{-j2p\pi m/{\bf{P}}},$$

where *p* represents frequency and ${\rm{\Xi }}$ represents the used Hamming window which is expressed as

42 $${\rm{\Xi }}(o)=\alpha -\beta \,\cos (\frac{2\pi o}{O}),\,o=0,\,1,\,\cdots ,\,O,$$

where *α* = 0.54 and *β* = 0.46. The DSFT window length must be chosen carefully. If it is too small, there will be excessive, and the result tends to zero length increases. A window of *O* = 512 was chosen based on extensive simulation results. The DSFT is shown in Fig. 6 and has a range error of only 0.104 m. Figure 7 shows the calculated SD values and the corresponding spectrums using DSFT without a human subject in the environment. Compared Figs 6 with 7(b), result indicates that the range information can be acquired by employing the DSFT technique, which is given by

43 $$\widehat{L}=\frac{v\widehat{\tau }}{{\rm{2}}}$$

where $\widehat{\tau }$ denotes the time delay, i.e. the peak value from (39).

### Frequency Estimation

The time index for the delay estimate $\widehat{\tau }$ is

44 $$\Im =\widehat{\tau }/{\delta }_{T}.$$

To estimate the vital sign signal frequencies, an FT is performed on (39), which gives

45 $$Y(\upsilon ,\,{\rm{t}})={a}_{v}S(\upsilon ){e}^{-j2\pi \upsilon {\tau }_{v}(t)}.$$

Using the time index in (44), (45) can be given by

46 $$Y(\Im ,\,{\rm{t}})={a}_{v}S(\frac{\Im }{{\delta }_{{\rm{T}}}}){e}^{-j2\pi \frac{\Im }{{\delta }_{{\rm{T}}}}{\tau }_{v}(t)},$$

and taking the logarithm gives^4^

47 $$ln[Y(\Im ,\,t)]=ln({a}_{v})+ln[S(\frac{\Im }{{\delta }_{{\rm{T}}}})]-j2\pi \frac{\Im }{{\delta }_{{\rm{T}}}}{\tau }_{v}(t)+j2k\pi ,$$

where the term *j*2*kπ* represents the phase variation due to any non-static objects and *k* is an integer. The unwrapping method can be used to remove this term^20^, but differentiation provides a simpler solution^16^. The imaginary part of (47) includes the phase variation of the vital signs $j2\pi \frac{\Im }{{\delta }_{{\rm{T}}}}{\tau }_{v}(t)$. Compared with the approach in Section II, the products of the vital signs and the harmonics are not as significant when only the phase is considered. Further, differentiating the logarithm term is simple and straightforward, making this method suitable for real-time vital sign detection^16^. Using (3), the result is

48 $$\lambda \text{'}=4{\pi }^{2}\frac{\Im }{{\delta }_{{\rm{T}}}}[{f}_{r}{\tau }_{r}\,\cos (2\pi {f}_{r}t)-{f}_{h}{\tau }_{h}\,\sin (2\pi {f}_{h}t)]$$

where $\text{'}$ denotes differentiation, and the corresponding discrete form as

49 $$\lambda \text{'}[{\rm{n}}]=4{\pi }^{2}\frac{\Im }{{\delta }_{{\rm{T}}}}[{f}_{r}{\tau }_{r}\,\cos (2\pi {f}_{r}n{T}_{s})-{f}_{h}{\tau }_{h}\,\sin (2\pi {f}_{h}n{T}_{s})]$$

The respiration frequency is typically 0.2 Hz to 0.5 Hz with an amplitude of 5 to 15 mm, and the heart rate is 0.8 Hz to 2.5 Hz with an amplitude of 2 to 3 mm^38^. The SNCR can thus be improved using a window for these frequencies. A rectangular window with length $\kappa$ in the frequency domain is employed for each slow-time dimension which gives

50 $${\boldsymbol{\Sigma }}[n]=\chi [n]\odot {\rm{FFT}}\{\lambda \text{'}[n]\},\,n={k}^{\ast },\,{k}^{\ast }+1,\,\ldots ,\,{k}^{\ast }+\kappa -1$$

where $\odot$ is the window operator and ${k}^{\ast }$ is the minimum index. The FA method proposed in^48^ is employed to suppress any remaining clutters existing in vital sign signals in the same frequency band. This gives

51 $$\mathop{{\bf{a}}}\limits^{{\boldsymbol{a}}}[n]=l[n]+jl[n]$$

where

52 $$l[n]=\{\begin{array}{ll}2{\rm{\Sigma }}[n], & n > 0\\ {\rm{\Sigma }}[n], & n=0\\ 0, & n < 0\end{array}$$

## Performance Results

Compared with several well-known techniques, the detection capability of the presented algorithm is tested in this section. This is achieved using experimental results obtained in both indoor and outdoor environments.

### Experimental Setup

**Figure 8:**
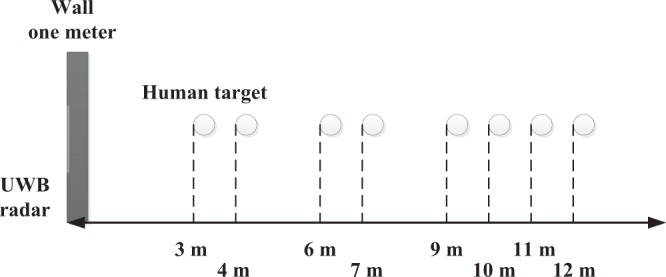
The UWB radar experimental setup.

Figure 8 shows the UWB radar experimental setup. The wall has a thickness of 100 cm and is composed of brick (30 cm), reinforced concrete (40 cm), and wood (30 cm). The four experiments described below were conducted to obtain data for performance evaluation.

**Figure 9:**
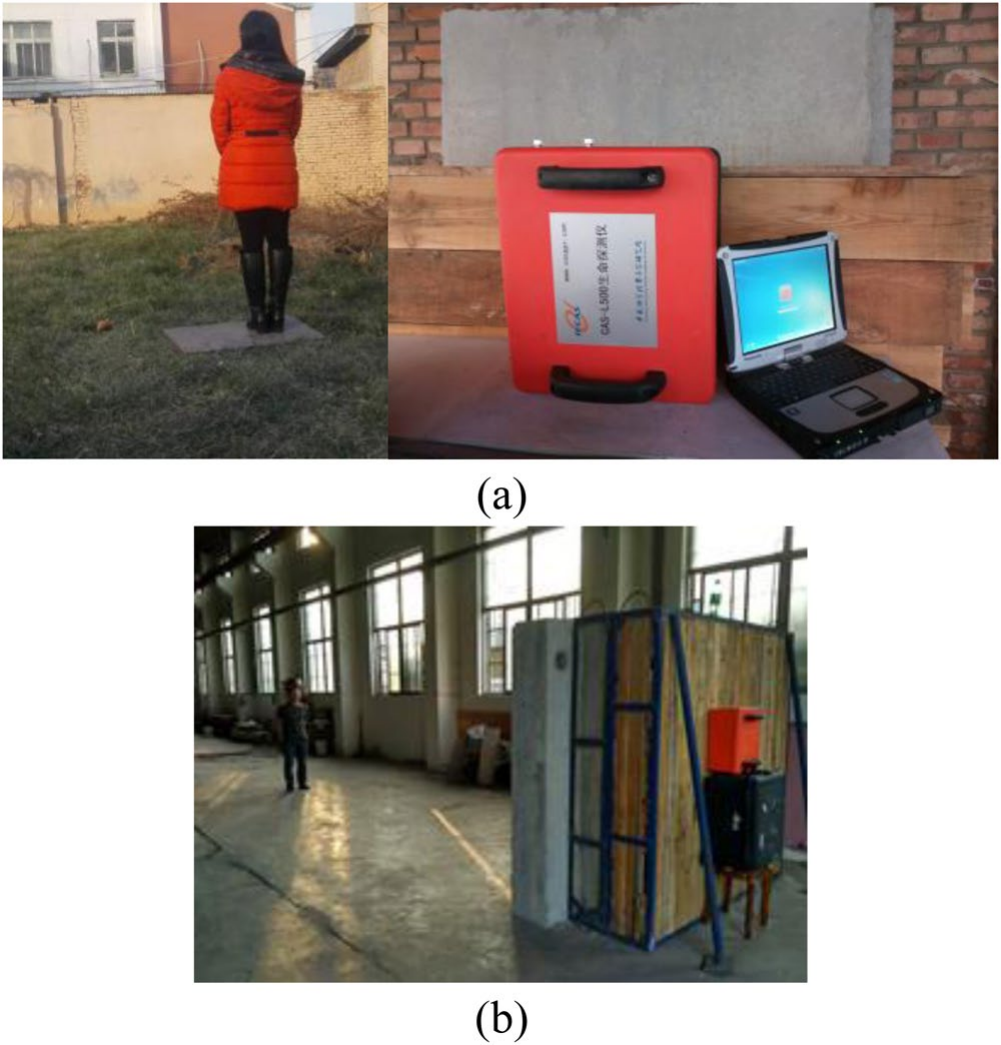
The experimental environment for a human subject (**a**) outdoors, and (**b**) indoors.

**Figure 10:**
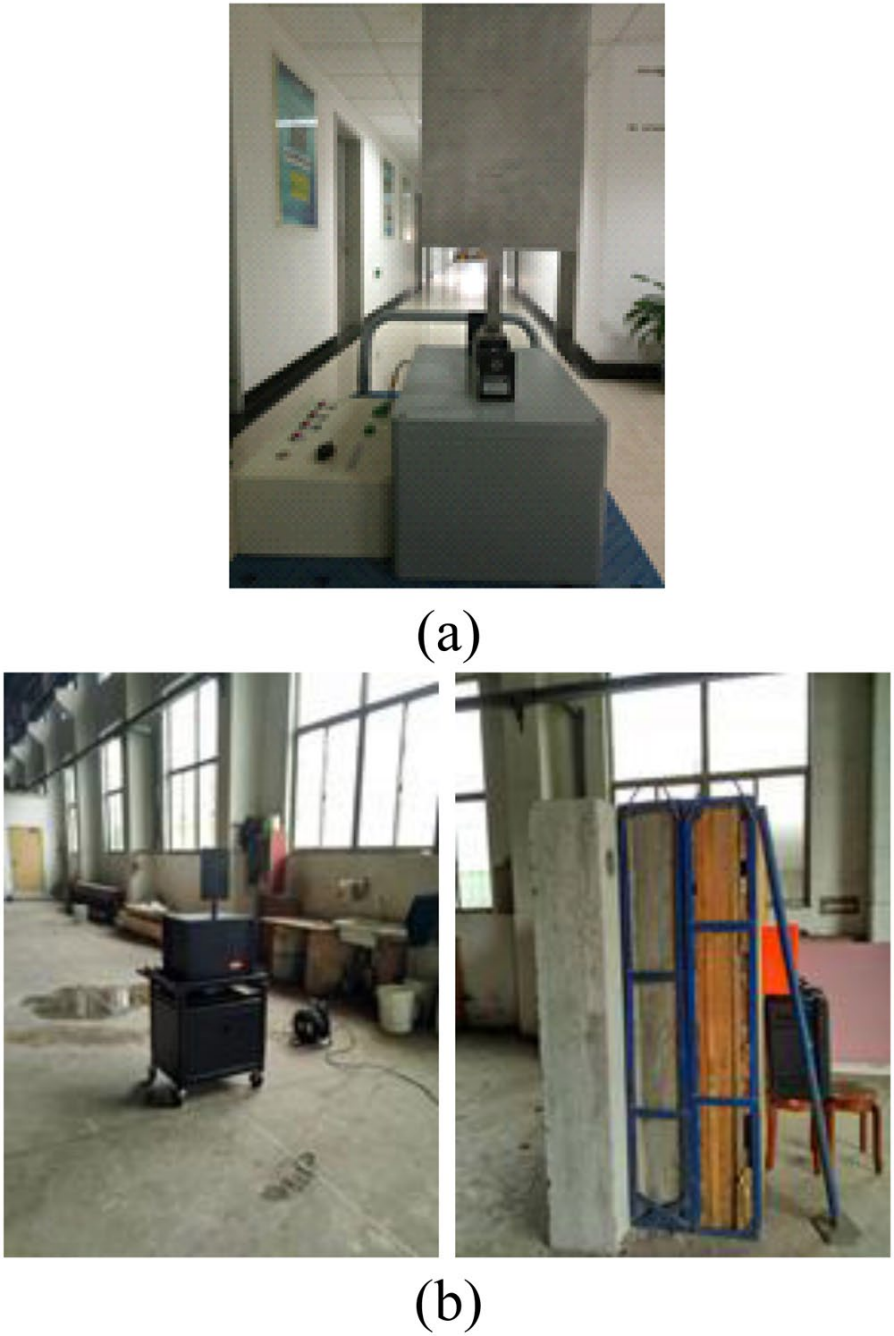
The actuator experiment (**a**) the actuator, and (**b**) the indoor environment.

1. This experiment was carried out at IECAS outdoors as shown in Fig. 9(a). A female subject stood breathing normally and facing the radar. The subject stood at 11 m, 9 m, 6 m, and 3 m far from the radar successively. The radar was placed on a table at a height of 1.5 m.
2. This experiment was conducted indoors at the China National Fire Equipment Quality Supervision Center (CNFEQSC) as shown in Fig. 9(b). A male subject stood 12 m, 10 m, 7 m, and 4 m far from the radar. The radar was placed on a table at a height of 1.3 m.
  An actuator was used instead of a human subject in experiments 3 and 4. The actuator moves at a frequency of 0.3333 Hz with an amplitude of 3 mm.
3. This experiment was carried out at IECAS outdoors as shown in Fig. 10(a). The actuator was placed on a table at a height of 1.3 m at a distance of 11 m from the radar.
4. This experiment was conducted at CNFEQSC indoors as shown in Fig. 10(b). The actuator was placed 12 m, 10 m, 7 m, and 4 m far from the radar.

The performance of the proposed, FFT, constant false alarm ratio (CFAR)^34^, and MHOC^37^ methods as well as the advanced method (AM)^36^, the FA method with different times, and the CSD^42^ and logarithm CSD (LCSD) techniques are evaluated in the following sections using the experimental data.

### Initial Detection Performance

**Figure 11:**
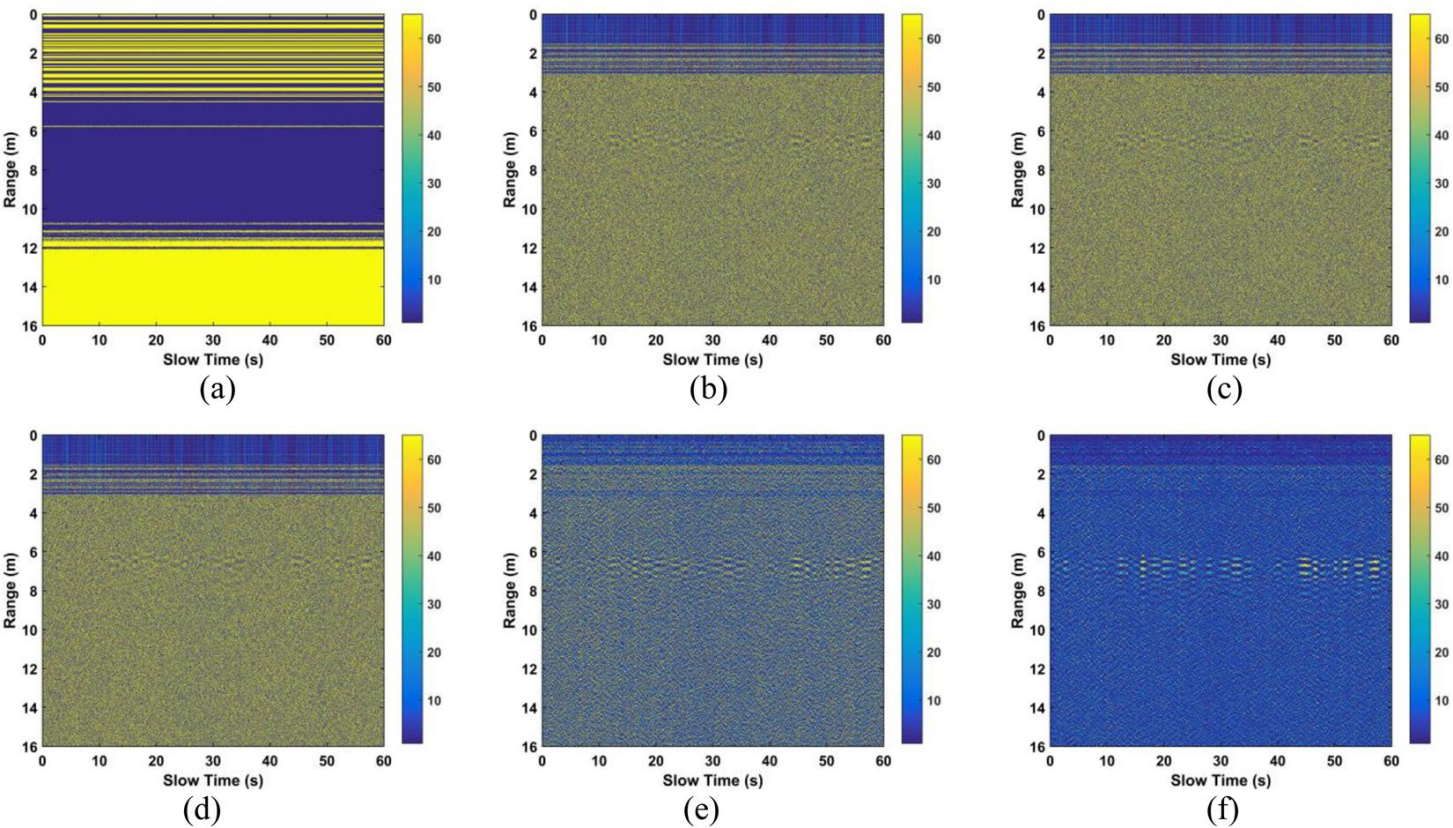
The results after (**a**) TMS, (**b**) removing the stationary clutter, (**c**) LTS, (**d**) SVD, (**e**) filtering in fast-time, and (**f**) filtering in slow-time.

In this subsection, based on the data from experiment 1, the performance of the clutter suppression steps is considered with the subject 9 m from the radar. The results after removing the DC component and static clutter are given in Fig. 11(a,b), respectively, and Fig. 11(c) presents the results after removing the linear trend. These figures show that human respiration signals are quite weak and as a consequence, the oscillations are challenging to determine. The results after SVD and fast-time filtering are given in Fig. 11(d,e), respectively. These show a further reduction in the clutter which improves the respiration signal. Figure 11(f) shows the results after slow-time filtering and indicates that the respiration signal is significantly improved compared to the received signal in Fig. 2.

### Human Subject Detection Outdoors

In this subsection, the data from experiment 1 is used for evaluating the performance of the detection method. Usually, the SNR of vital signs increases with decreasing distance between the radar and human subject due to large-scale attenuation^32^. The improvement in SNR can thus be used to evaluate the performance, which can be obtained as

53 $$SNR=20\,{\mathrm{log}}_{10}(\frac{\sum _{n={\mu }_{r}-1}^{{\mu }_{r}+1}|\mathop{{\bf{a}}}\limits^{{\boldsymbol{a}}}[n]|}{\sum _{n={\gamma }_{1}}^{{\mu }_{r}-2}|\mathop{{\bf{a}}}\limits^{{\boldsymbol{a}}}[n]|+\sum _{n={\mu }_{r}+2}^{{\gamma }_{2}}|\mathop{{\bf{a}}}\limits^{{\boldsymbol{a}}}[n]|}),$$

where ${\mu }_{r}$ is the frequency index corresponding to the target, and ${\gamma }_{1}$ and ${\gamma }_{2}$ are the boundaries of the frequency window.

**Figure 12:**
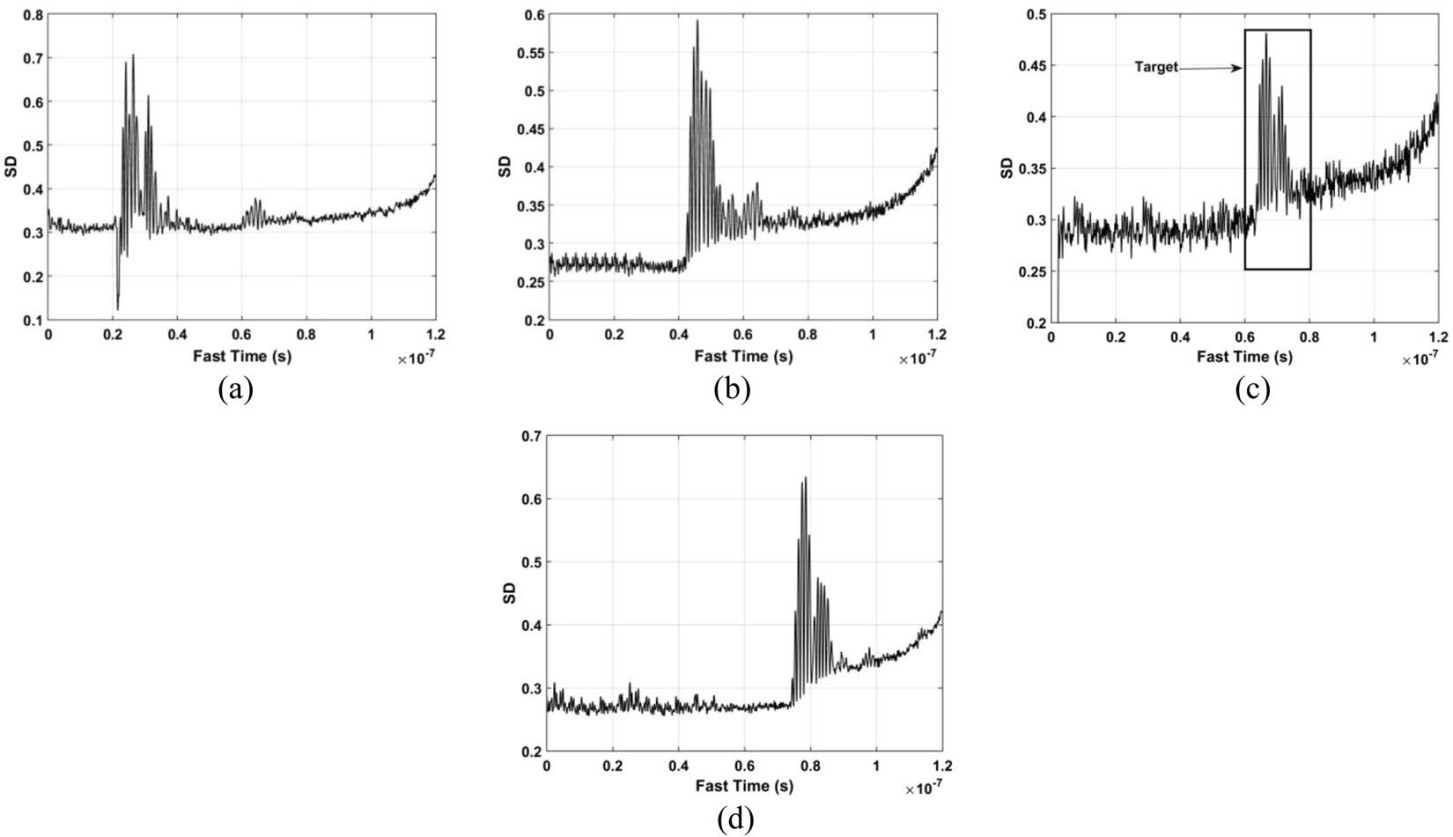
The SD for a subject outdoors located (**a**) 3 m, (**b**) 6 m, (**c**) 9 m, and (**d**) 11 m from the radar.

**Figure 13:**
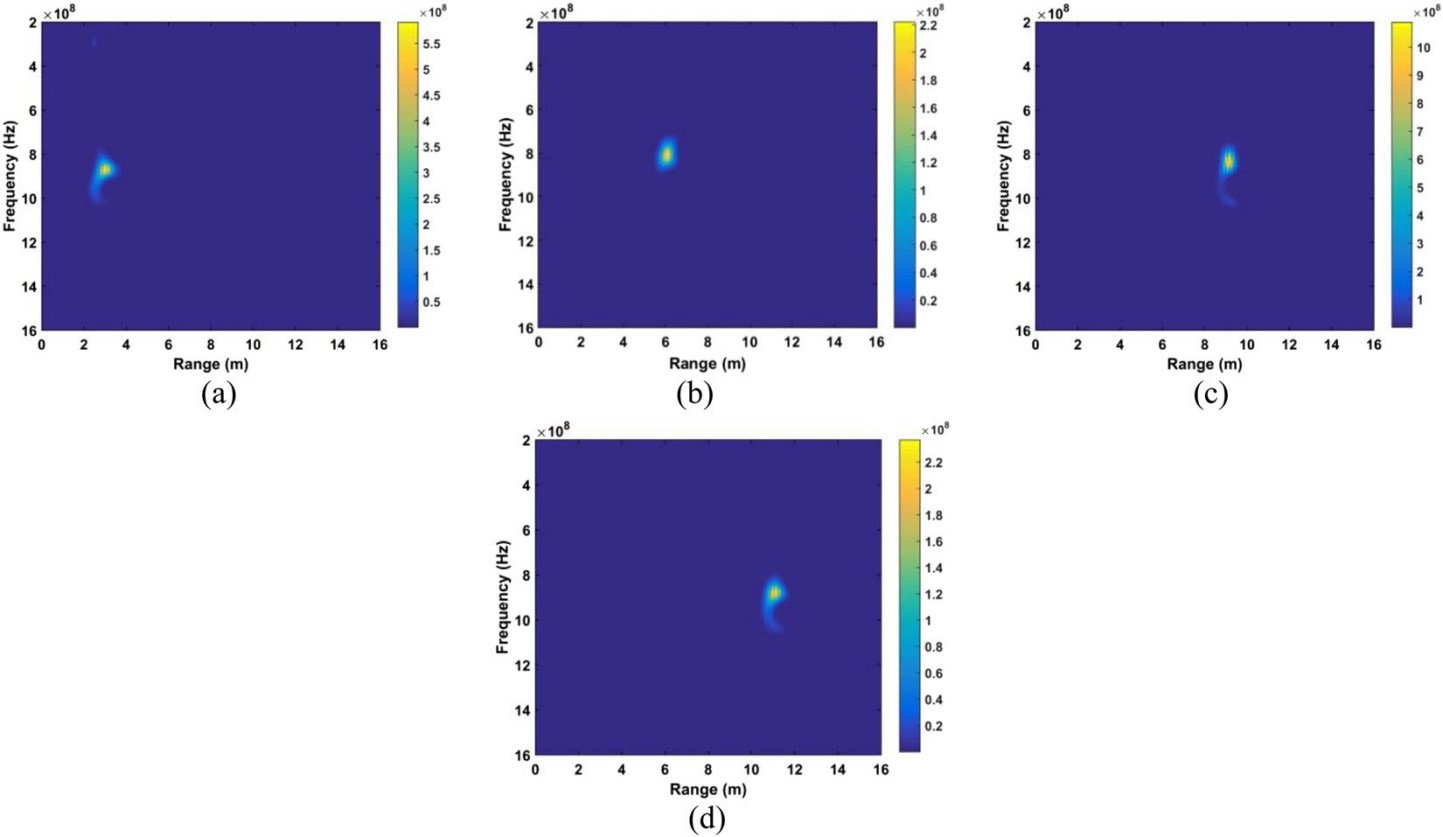
Range estimation using an STFT for a subject located (**a**) 3 m, (**b**) 6 m, (**c**) 9 m, and (**d**) 11 m from the radar.

The data sets acquired outdoors were used to evaluate four detection methods. Figure 12 gives the calculated SD results and the corresponding spectrums using the DSFT technique are shown in Fig. 13. The errors in the estimations of range information are 26 cm, 11 cm, 5 cm, and 5 cm at ranges of 11 m, 9 m, 6 m and 3 m, respectively.

**Figure 14:**
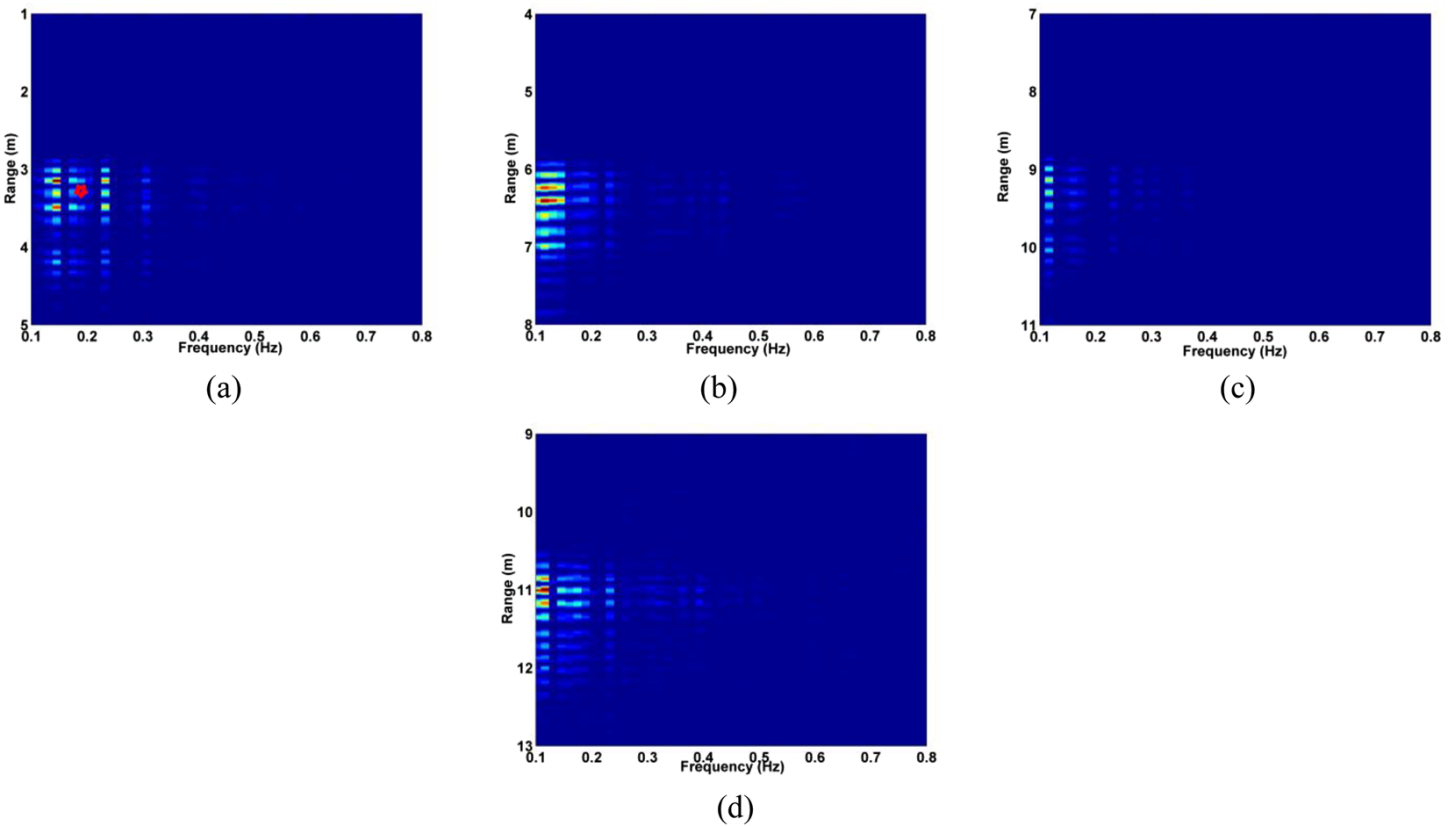
The range estimation using the CFAR method for a subject located (**a**) 3 m, (**b**) 6 m, (**c**) 9 m, and (**d**) 11 m from the radar.

Table 1 gives the estimations of human respiration frequency, range errors, and SNR values from (53) for four detection algorithms. This shows that the presented method supplies the most accurate range estimates and high SNR, particularly for long distances. At a distance of 11 m, the SNR with the proposed method is 3.25 dB while the SNR is only −15.3 dB with the CFAR method, a difference of 18.6 dB. The respiration frequency estimates using the proposed method are 0.26 Hz at 11 m, 0.29 Hz at 9 m, 0.26 Hz at 6 m, and 0.32 Hz at 3 m, respectively, which are considerably more accurate than with the other methods. Figure 14 gives the results acquired from the CFAR technique, which indicate that the range information cannot be estimated accurately with this method over long distances. Figure 14(b–d) show that there remains significant clutter at distances of 6 m or more.

**Table 1:** Vital sign estimation with four methods.

Algorithm		Rang (m)			
		11	9	6	3
Proposed	Error (cm)	20	11	5	5
	Rate (Hz)	0.26	0.29	0.26	0.32
	SNR (dB)	3.25	3.67	4.32	7.58
CFAR	Error (cm)	954	672	436	27
	Rate (Hz)	0.46	0.72	0.10	0.18
	SNR (dB)	−15.3	−11.9	−7.22	−4.54
AM	Error (cm)	398	467	546	24
	Rate (Hz)	0.63	0.74	0.12	0.37
	SNR (dB)	−6.59	−3.69	0.84	5.35
MHOC	Error (cm)	725	156	243	35
	Rate (Hz)	0.44	0.52	0.45	0.14
	SNR (dB)	−11.4	−8.58	−5.85	−2.67

**Figure 15:**
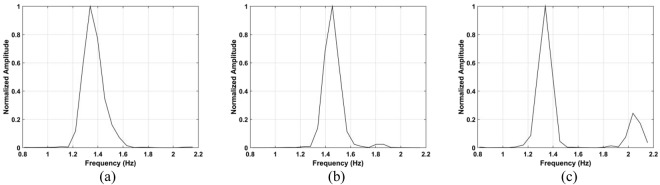
Heartbeat frequency estimation using the proposed method for a subject located (**a**) 3 m, (**b**) 6 m, and (**c**) 9 m from the radar.

To evaluate the heart rate estimation performance, the data with a male subject outdoors was used. A typical resting heart rate is 75 to 81 beats per minute, corresponding to frequencies of 1.25 to 1.35 Hz. Figure 15 shows that the heart rate estimates for the proposed method are 1.34 Hz, 1.34 Hz, and 1.45 Hz, respectively. It was not possible to obtain estimates using the CFAR method. The corresponding SNR values for the proposed and CFAR methods are given in Table 2. This shows that the proposed method provides a significant SNR improvement over the CFAR method as the smallest difference is 9.43 dB at a distance of 6 m. The very low SNR values for the CFAR method are the reason the heart rate could not be estimated.

**Table 2:** Heart rate detection SNR (dB) for the proposed and CFAR methods.

Method	3 m	6 m	9 m
Proposed	6.57	4.22	1.23
CFAR	−3.56	−5.21	−12.6

### Human Subject Detection Indoors

**Figure 16:**
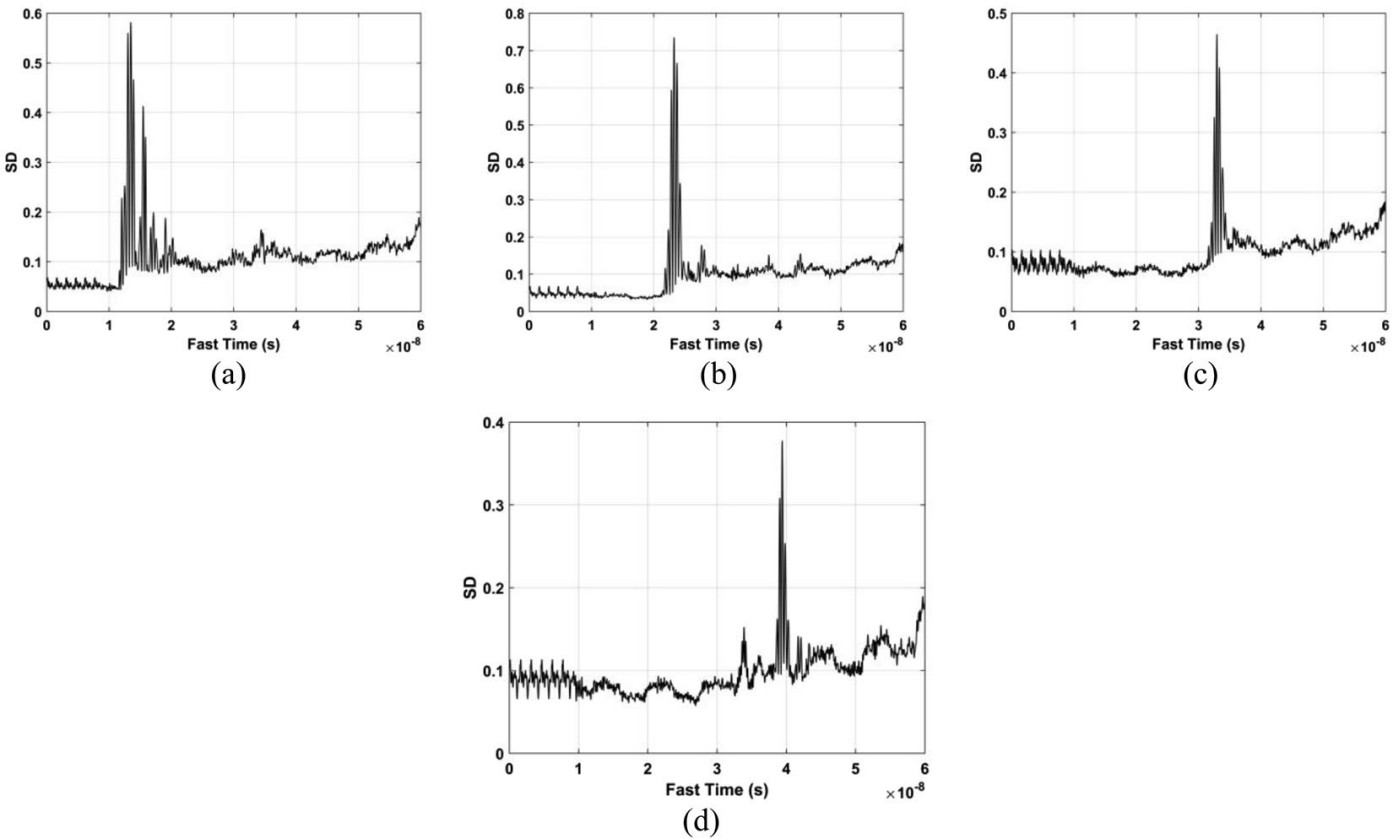
The SD for a subject indoors located (**a**) 4 m, (**b**) 7 m, (**c**) 10 m, and (**d**) 12 m from the radar.

**Figure 17:**
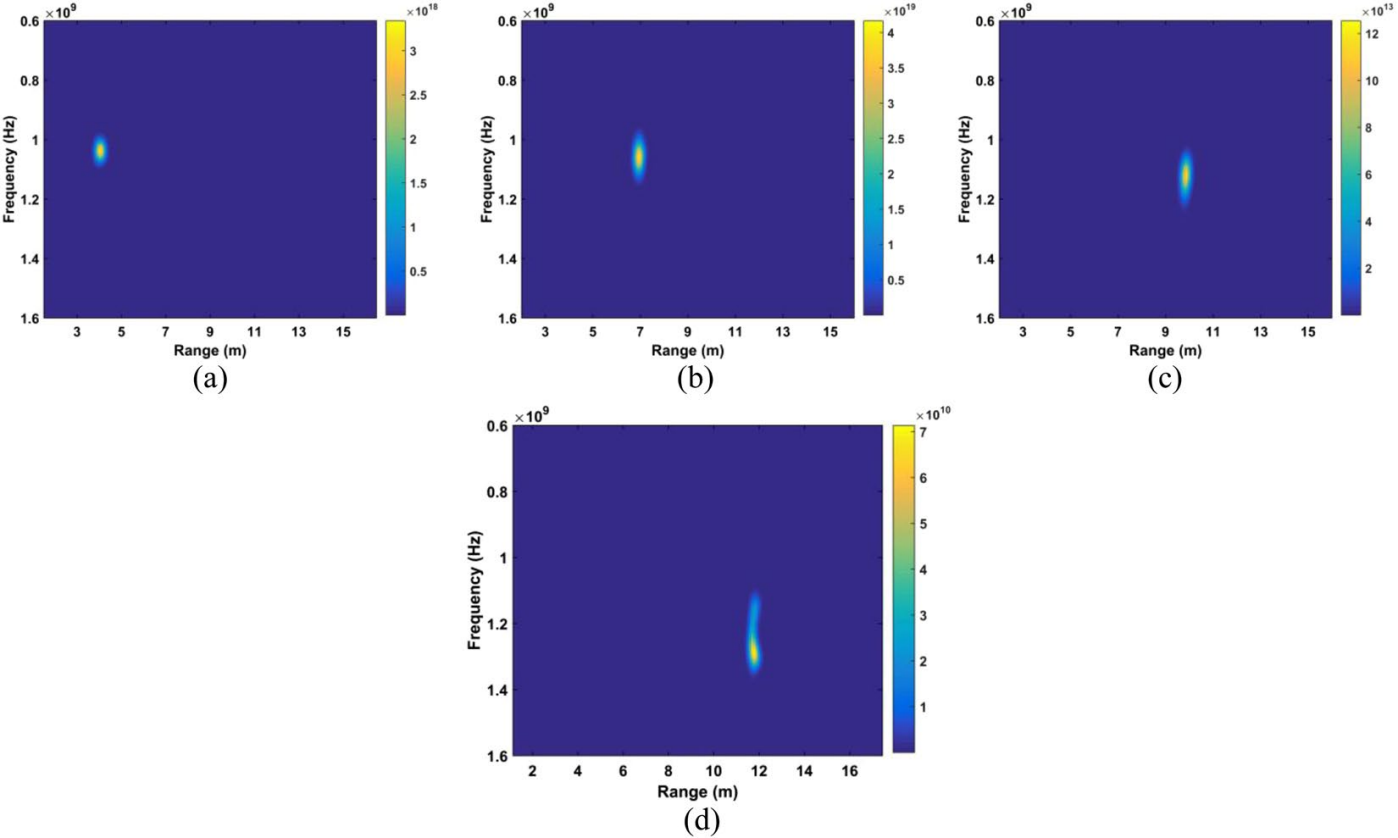
Range estimation using and STFT for a subject indoors located (**a**) 4 m, (**b**) 7 m, (**c**) 10 m, and (**d**) 12 m from the radar.

**Figure 18:**
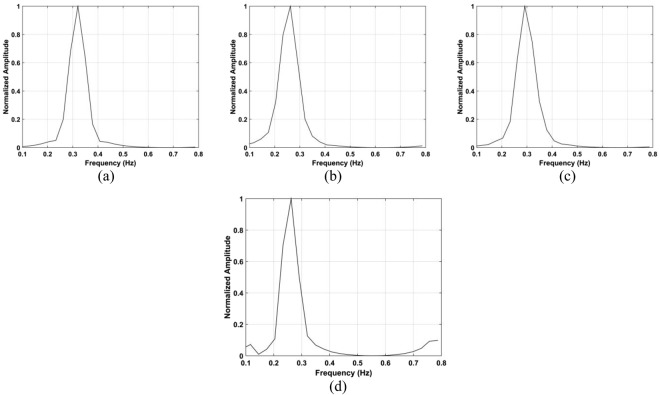
Respiration frequency estimation using the proposed method for a subject located (**a**) 4 m, (**b**) 7 m, (**c**) 10 m, and (**d**) 12 m from the radar.

The data from experiment 2 is now used for evaluating the detection performance. Figure 16 shows the calculated SD values with the proposed method and the DSFT results are given in Fig. 17. The range estimates are 11.8 m, 9.89 m, 6.95 m, and 4.06 m for distances of 12 m, 10 m, 7 m and 4 m, respectively, and show that the range information can be estimated more accurately indoors. This is because it is a controlled environment which lacks factors such as wind. The respiration frequency estimation results are shown in Fig. 18. The estimates of for the four distances are 0.26 Hz, 0.29 Hz, 0.26 Hz, and 0.32 Hz.

### Clutter Suppression

**Figure 19:**
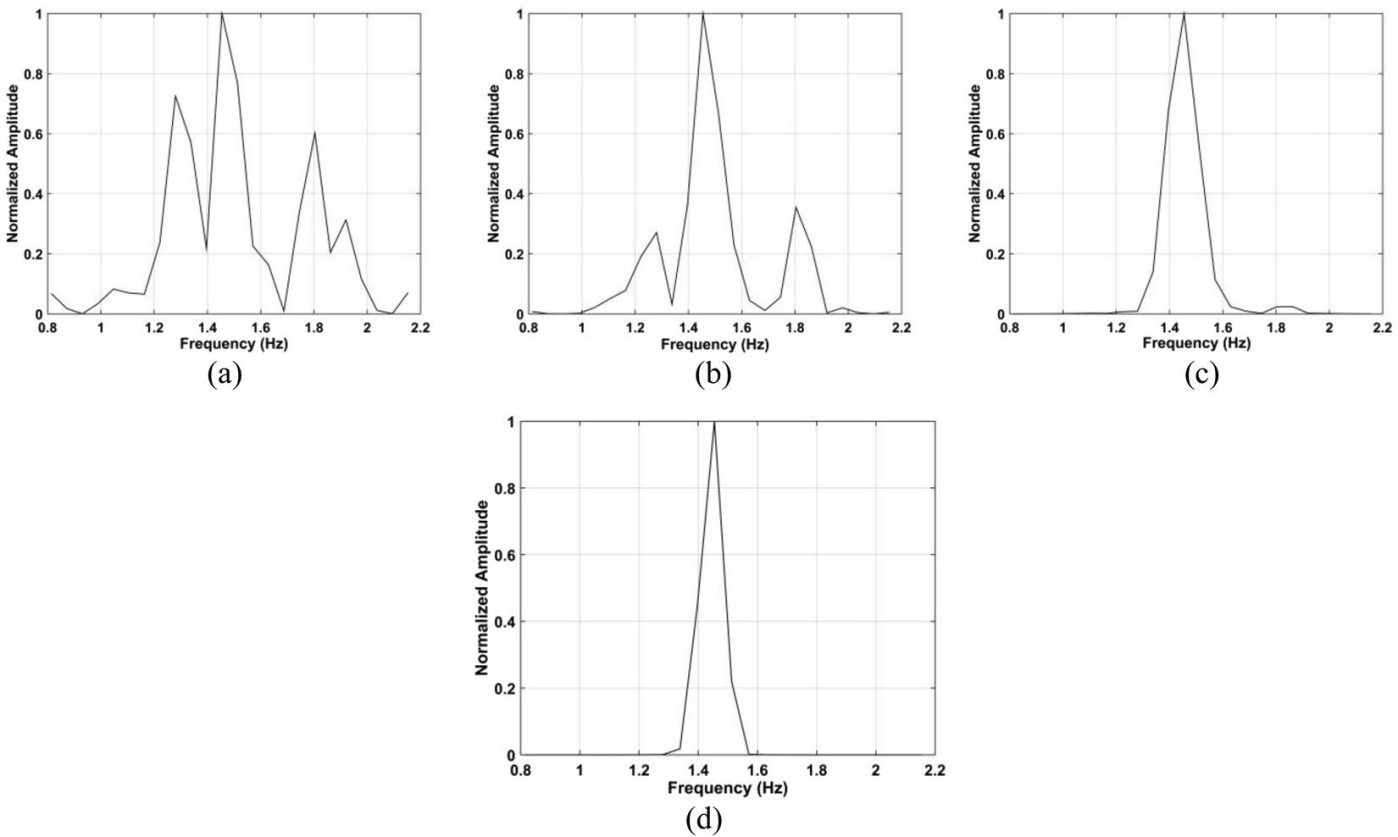
Clutter suppression results using the FA method (**a**) once, (**b**) twice, (**c**) four times, and (**d**) six times.

**Figure 20:**
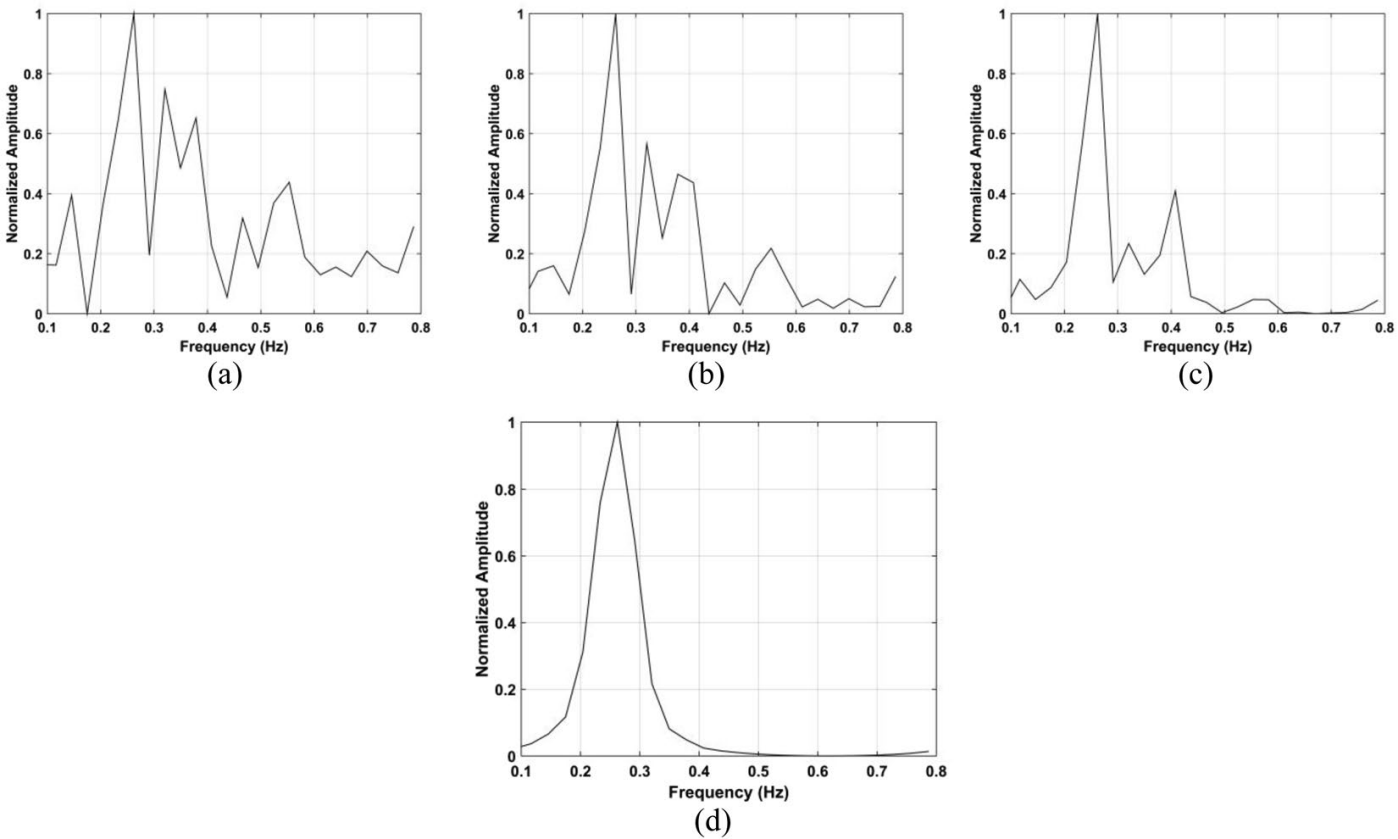
Clutter suppression results using the (**a**) FFT, (**b**) CSD-based one-FA, (**c**) CSD-based two-FA, and (**d**) CSD-based four-FA methods.

**Figure 21:**
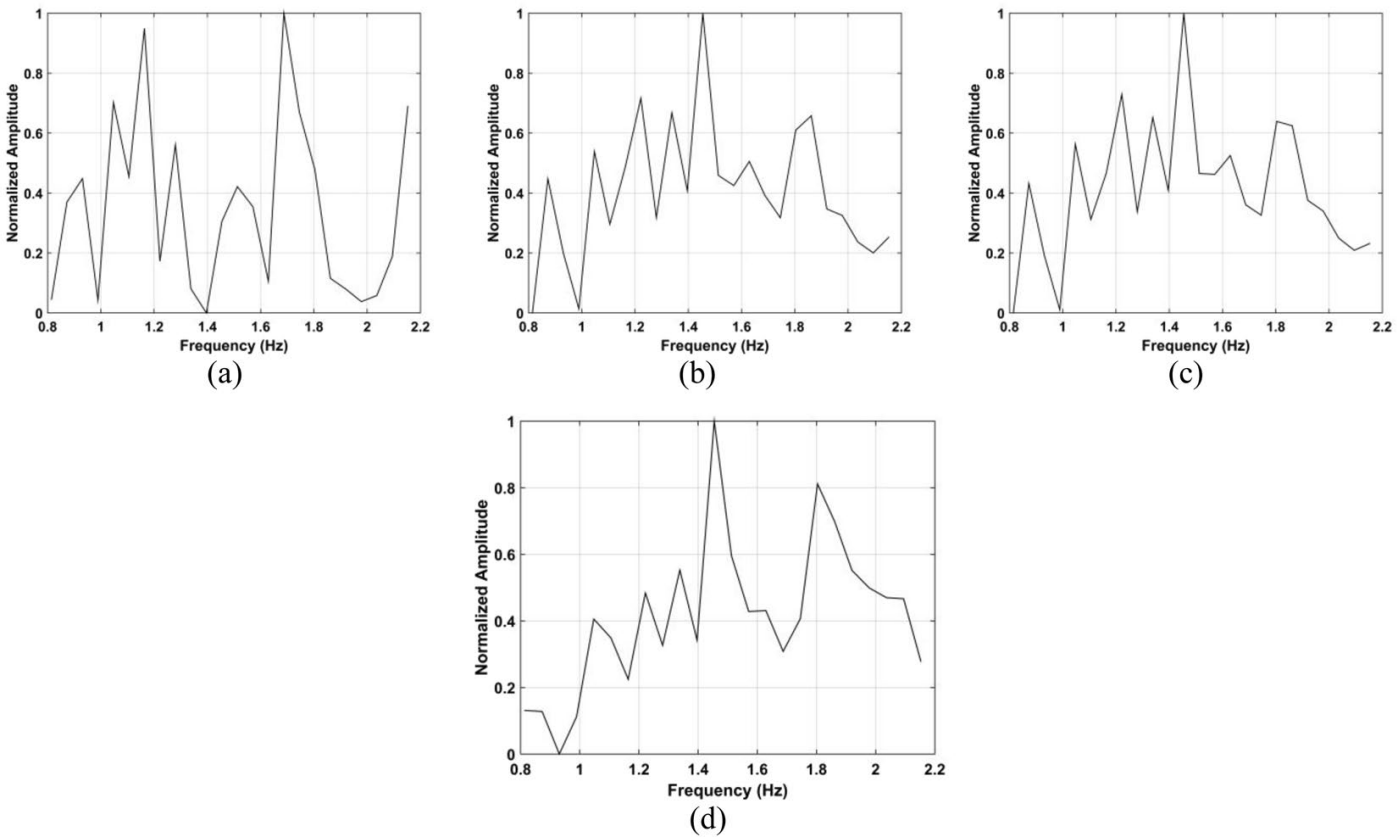
Clutter suppression results using the (**a**) FFT, (**b**) CSD, (**c**) LCSD, and (**d**) DLCSD methods.

The capability of removing clutters was evaluated using the data from experiment 1 at 6 m. The harmonics and the products of the vital sign signals were suppressed using the FA method and the results are given in Figs 19 and 20. This shows that the four-FA method can supply a better SNR improvement than the FFT, two-FA, and one-FA methods. Table 3 gives the SNR values and shows that the additional improvement is minimal when the FA method is performed six times. The SNR for the FFT, CSD, logarithm CSD (LCSD) and differentiated LCSD (DLCSD) methods is also given in Table 4. The corresponding frequency domain results for the CSD, LCSD, and DLCSD methods are given in Fig. 21(b–d), respectively. The range estimation results in Fig. 14 show that these methods do not effectively suppress the clutter compared to the proposed method.

**Table 3:** The SNR improvement with eight methods.

Method	SNR (dB)
FFT	0.39
CSD	0.23
LCSD	0.23
DLCSD	0.24
One FA	0.50
Two FA	1.26
Four FA	4.13
Six FA	4.36

**Table 4:** Frequency estimates with four methods.

Method	1	2	3	Deviation (%)
FFT	0.29	0.11	0.23	36
CSD	0.29	0.46	0.11	39
LCSD	0.40	0.17	0.34	25
Proposed	0.34	0.34	0.34	4.7

### Actuator Experiment Results

**Figure 22:**
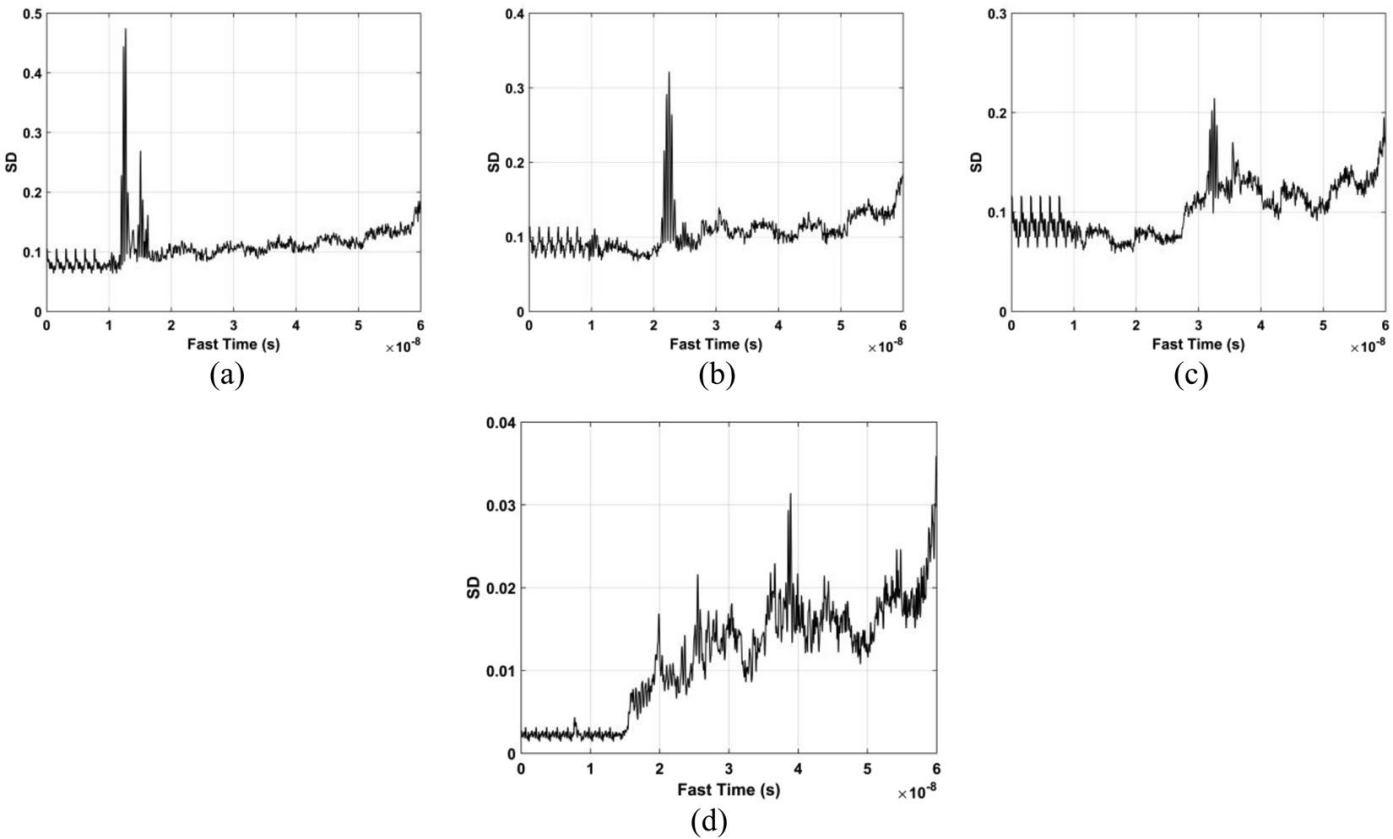
The SD with the actuator indoors located (**a**) 4 m, (**b**) 7 m, (**c**) 10 m, and (**d**) 12 m from the radar.

**Figure 23:**
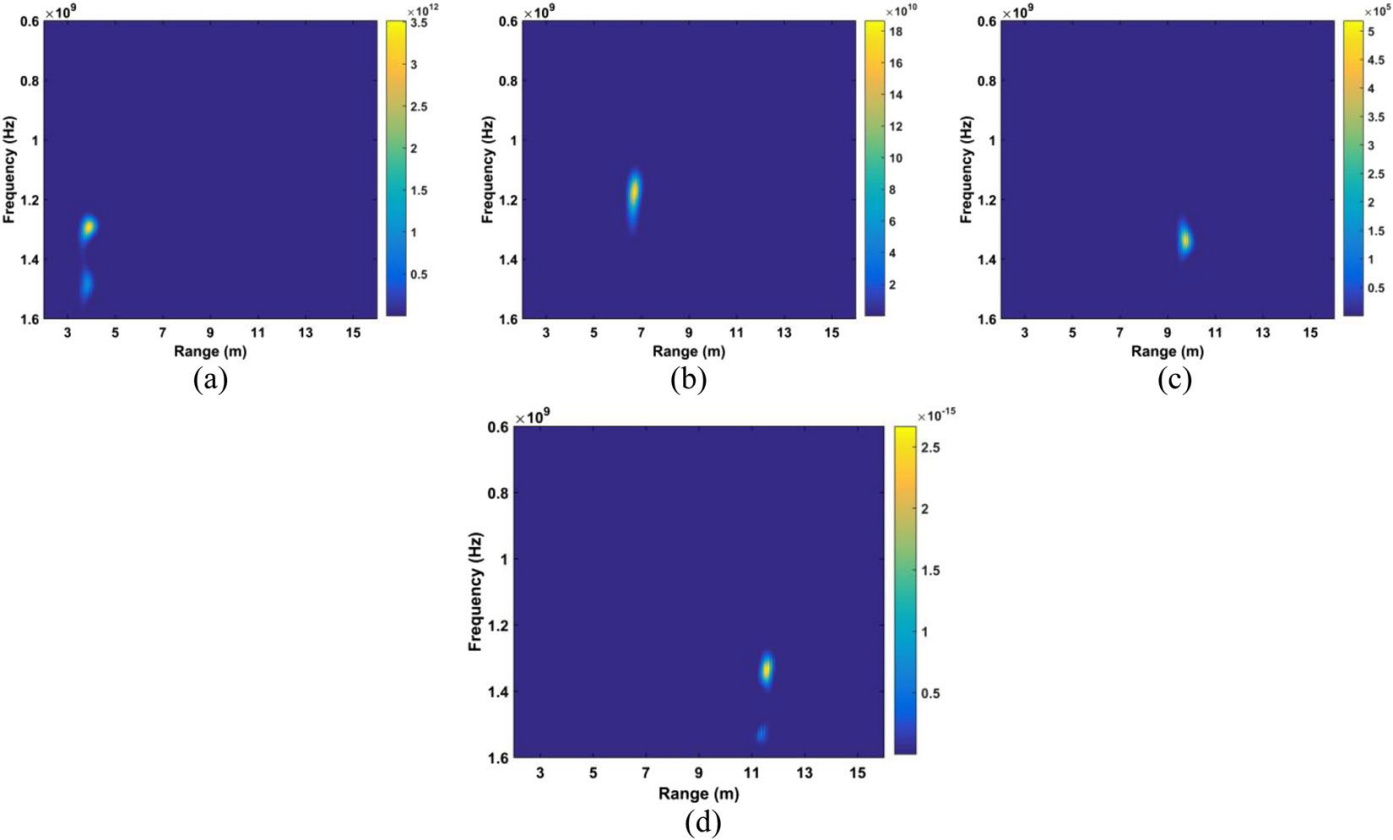
Range estimation using the proposed method with the actuator located (**a**) 4 m, (**b**) 7 m, (**c**) 10 m, and (**d**) 12 m from the radar.

**Figure 24:**
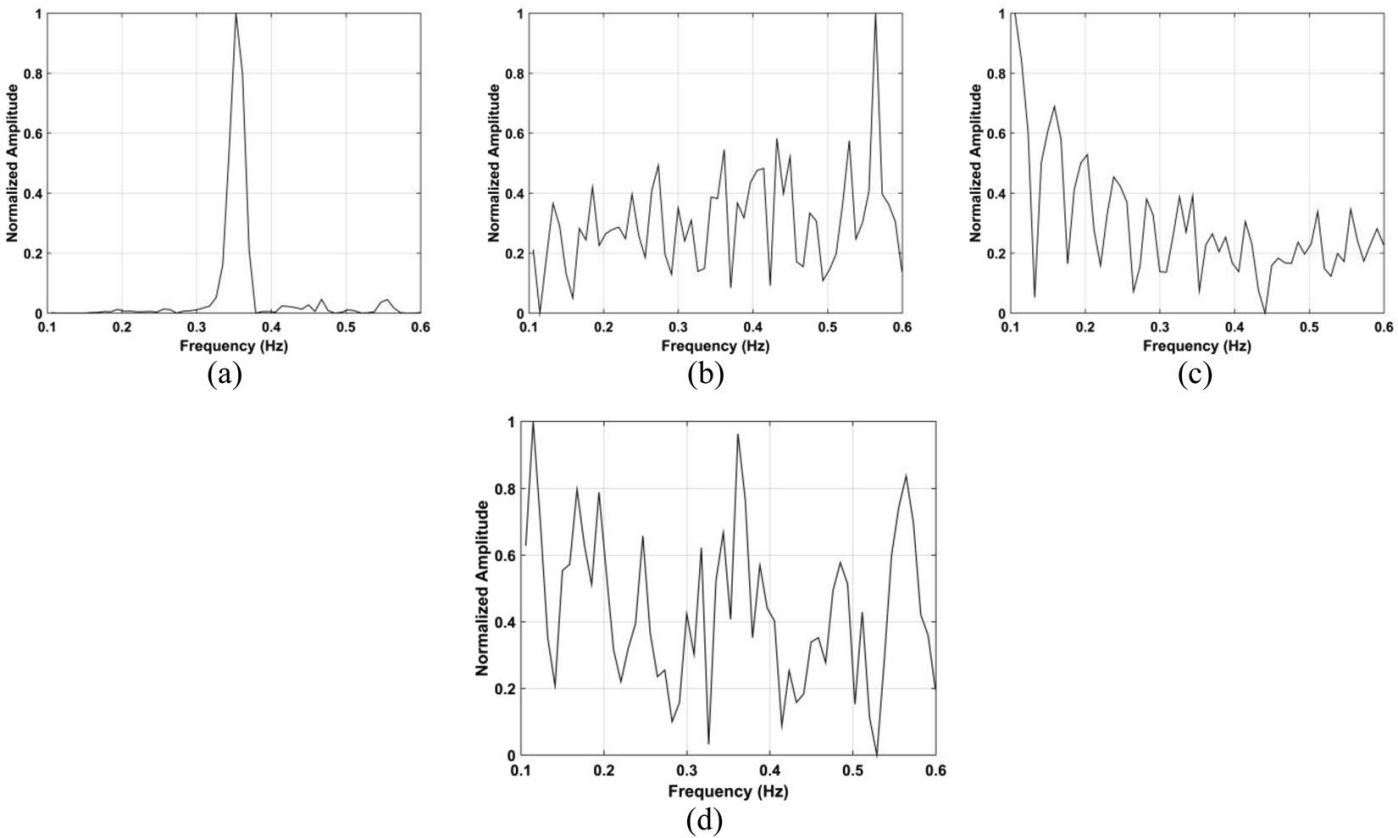
Actuator frequency estimation using the (**a**) proposed, (**b**) CSD, (**c**) LCSD, and (**d**) FFT methods.

**Figure 25:**
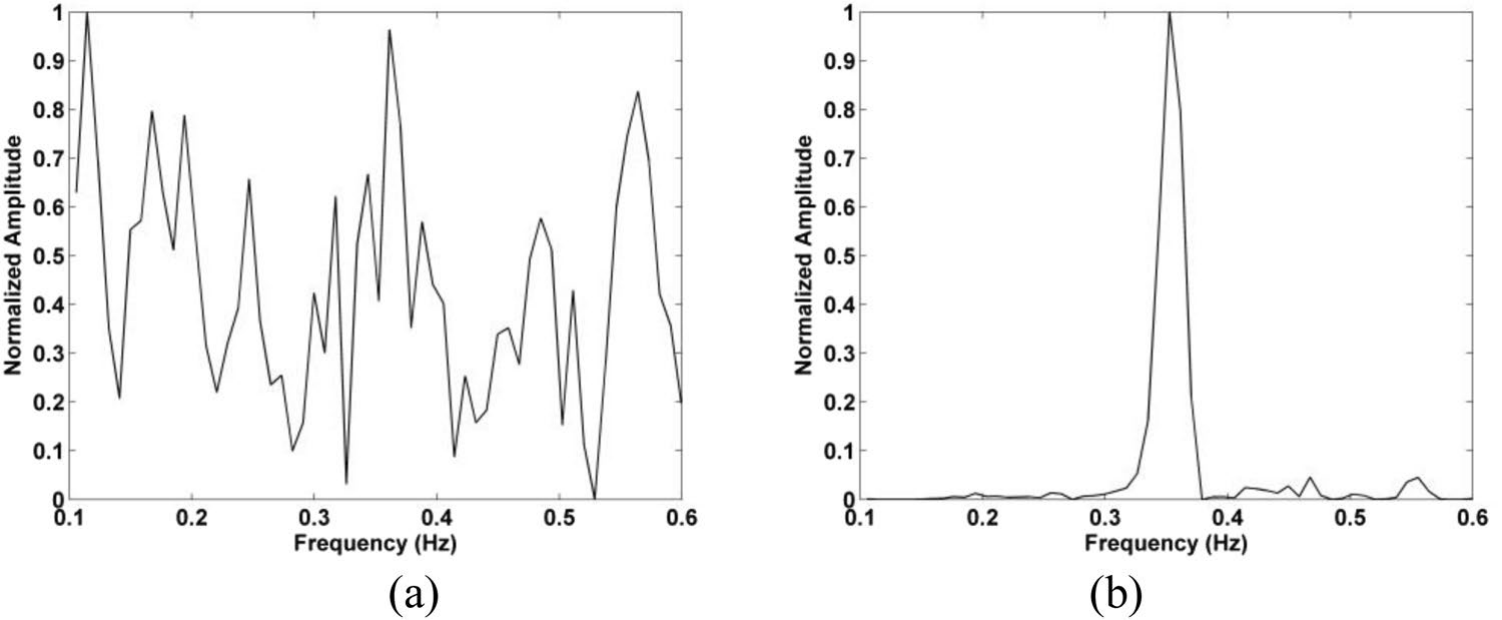
Frequency estimation with the actuator located 11 m from the radar using the (**a**) FFT and (**b**) proposed methods.

The data from the conducted actuator experiments were used for testing the presented algorithm. The actuator was used to imitate human respiration with the amplitude of 3 mm and a frequency of 0.3333 Hz and. Figure 22 shows the SD values for the proposed method using the data from experiment 4 and the corresponding range and frequency estimation results are given in Figs 23 and 24, respectively. The frequency estimate from Fig. 24(a) is 0.353 Hz. The frequency estimate using the CSD method from Fig. 24(b) is 0.11 Hz and the estimate using the LCSD method from Fig. 24(c) is 0.56 Hz. The FFT method provides the worst performance as the frequency estimate from Fig. 24(d) is 0.12 Hz. Compared with the actual frequency of 0.3333 Hz, the proposed method provides the best estimate with an error of only 0.019 Hz. The frequency estimates using the outdoor data from experiment iii is shown in Fig. 25. The frequency estimates and corresponding deviations are given in Table 4 for three trials of the experiment. All these results indicate that the presented algorithm has the smallest deviation and significantly outperforms the other algorithms.

## Conclusion

Vital sign detection was considered in this paper for applications such as after a natural disaster. The respiration and heartbeat frequencies were estimated using an impulse UWB radar. The CSD-based FA method was shown to be effective in suppressing signal products and harmonics. The discrete short-time Fourier transform (DSFT) of the calculated standard deviation (SD) values was used to estimate the range information of the volunteer. Experimental data obtained using a UWB radar were used to evaluate the performance of several techniques. The results obtained indicate that the proposed method can more effectively remove clutter and improve the SNR than the other methods. Further, it provides better range and frequency estimates^49–53^.

## Data Availability

All data analyzed during this study are included in this paper.
